# Sutureless vs. rapid-deployment valve: a systemic review and meta-analysis for a direct comparison of intraoperative performance and clinical outcomes

**DOI:** 10.3389/fcvm.2023.1123487

**Published:** 2023-05-15

**Authors:** Chenhao Wang, Yi Xie, Hongwei Zhang, Peng Yang, Yu Zhang, Chen Lu, Yu Liu, Haiyue Wang, Zhenyuan Xu, Jia Hu

**Affiliations:** ^1^ Department of Cardiovascular Surgery, West China Hospital, Sichuan University, Chengdu, Sichuan, China; ^2^ Cardiovascular Surgery Research Laboratory, West China Hospital, Sichuan University, Chengdu, Sichuan, China; ^3^ Department of Cardiovascular Surgery, Guang'an Hospital of West China Hospital, Sichuan University, Guang'an, Sichuan, China

**Keywords:** Perceval, Intuity elite, aortic valve replacement, pressure gradient, CPB (cardiopulmonary bypass), aortic cross clamp

## Abstract

**Background:**

Sutureless and rapid-deployment valves are bioprostheses anchoring within the aortic annulus with few sutures, and they act as a hybrid of conventional surgical and transcatheter valves under aortic valve replacement. Considering that the 3F Enable valve is now off-market, the only two sutureless and rapid-deployment valves available on the world marketplace are the Perceval and Intuity valves. However, a direct comparison of the function of these two valves eludes researchers.

**Purpose:**

Against this background, we performed this systematic review and meta-analysis comparing the intraoperative performance and early clinical outcomes between the Perceval valve and the Intuity valve under sutureless and rapid-deployment aortic valve replacement.

**Methods:**

We systematically searched electronic databases through PubMed/MEDLINE, OvidWeb, Web of Science, and Cochrane Central Register of Controlled Trials (from the establishment of the database to November 17, 2022, without language restriction) for studies comparing the sutureless valve (the Perceval) and the rapid-deployment valve (the Intuity) under aortic valve replacement. Our primary outcomes were early mortality and postoperative transvalvular pressure gradients. The secondary outcomes were defined to include aortic cross-clamp and cardiopulmonary bypass time, paravalvular leak (any paravalvular leak, moderate-to-severe paravalvular leak) after aortic valve replacement, need for pacemaker implantation, postoperative neurological events (stroke), and intensive care unit stay.

**Results:**

This meta-analysis included ten non-randomized trials with 3,526 patients enrolled (sutureless group = 1,772 and rapid-deployment group = 1,754). Quality assessments were performed, with the mean scores of the studies reading 6.90 (SD = 0.99) out of 9 according to the Newcastle–Ottawa Scale. Compared with rapid-deployment aortic valve replacement, sutureless aortic valve replacement was associated with higher mean and peak transvalvular pressure gradients postoperatively. In contrast, aortic cross-clamp and cardiopulmonary time were needed less in sutureless aortic valve replacement vs. rapid-deployment aortic valve replacement. There was no evidence of significant publication bias observed by the funnel plot and Egger's test.

**Conclusions:**

For postoperative hemodynamics, sutureless aortic valve replacement was associated with increased mean and peak transvalvular pressure gradients compared with rapid-deployment aortic valve replacement. In sharp contrast, sutureless aortic valve replacement significantly reduced the amount of time needed for fixing the aortic cross-clamp and the cardiopulmonary bypass procedure.

**Systematic Review Registration:**

https://www.crd.york.ac.uk/prospero/, identifier CRD42022343884.

## Introduction

Aortic stenosis (AS) will become one of the most common valvular heart diseases as the population ages and life expectancy increases (1). Surgical aortic valve replacement (SAVR) is always considered the golden standard for treating AS (2). However, considering the high surgical risks involved, more than 30% of patients with severely symptomatic AS do not undergo surgery in clinical practice. Transcatheter aortic valve implantation (TAVI) has become an effective alternative established for the purpose of providing less-invasive treatment. Nevertheless, a crucial limitation of TAVI is that it is almost impossible to remove all native valve cusps or a degenerated prosthesis (3–6).

Recently, sutureless and rapid-deployment valves have emerged as prospective substitutes for typical valves (2). These valves are biological prostheses anchoring within the aortic annulus with at most three sutures (7, 8). With sufficient radial force to allow annular implantation without sutures in a sutureless valve and the rapid-deployment valve system providing an innovative extended balloon structure requiring only three sutures for fast deployment, these valves facilitate minimally invasive surgery and complex intervention in annulus decalcification and degenerated valve removal. Evidence from the Sutureless and Rapid-Deployment Aortic Valve Replacement International Registry (SURD-IR) enrolling more than 4,500 patients suggests that SURD-AVR is a secure and efficacious substitute for the conventional aortic valve replacement procedure (7, 9, 10).

Three sutureless and rapid-deployment prosthesis valves have received CONFORMITE EUROPEENNE (CE) market approval: the Perceval S, the Intuity, and the 3f Enable. , The 3f Enable valve was recalled in 2014 most probably because of elevated migration risks (8). Sutureless valve Perceval and rapid-deployment valve Intuity are the only two representatives of valves in SURD-AVR, both of which function well in sutureless and rapid-deployment aortic implantation by reducing aortic cross-clamp and cardiopulmonary bypass time and delivering excellent hemodynamic results (10, 11). At the same time, a previous study demonstrated that SURD-AVR was associated with an increased rate of pacemaker implantation postoperatively compared with SAVR (12). However, there are limited published data directly comparing both promising devices, and most of these data are only observational and retrospective studies rather than randomized controlled trials or only small sample studies riddled with deficiencies.

In this study, we performed a systematic review and meta-analysis to evaluate the intraoperative performance and early clinical outcomes between the sutureless and the rapid-deployment aortic valve replacement methods.

## Methods

### Data source and search strategy

We searched Pubmed/Medline, Ovidweb, Web of Science, and the Cochrane Central Register of Controlled Trials (CENTRAL) for relevant articles, from the date of establishment of the database to November 17, 2022, in all languages, using a combination of main terms and MeSH terms such as “aortic valve[MeSH terms]” or “heart valve prosthesis[MeSH terms]” or “aortic valve replacement” or “aortic valve implantation” and “sutureless” or “Perceval” and “rapid deployment” or “Intuity”. Next, we performed a search for additional sources of information for the literature supplement, including Google Scholar and abstracts/presentations from major international cardiovascular-relevant conferences. Finally, the reference lists of relevant works of literature were also checked for the supplement. The complete retrieval strategy is presented in Supplementary Table S1.

### Study selection and data extraction

Two investigators (CW and YX) independently performed the study selection on the basis of predetermined selection criteria. Any discrepancy among the investigators was resolved by a third investigator (JH). After removing duplicates, we performed selection through two levels: the title and abstract of each searched study were screened for relevance as part of the first level, and a full-text analysis of the remaining studies was done for inclusion as the second level. Studies were considered eligible for inclusion in our systematic review and meta-analysis if they fulfilled the following criteria: (1) enrolled patients undergoing aortic valve replacement and who used both sutureless and rapid-deployment valves; (2) those who reported at least one primary outcome, defined as early mortality (30-day all-cause mortality and in-hospital mortality) and postoperative transvalvular pressure gradients (mean/peak); (3) the sample size of each group should be more than 10; (4) there should be no duplicated population figures across studies. Without any restrictions as full texts, abstract reports from important conferences that met the inclusion criteria were also considered in our study.

Using standardized data collection sheets that recorded essential items, we extracted the following data from each included study: study characteristics [publication characteristics (authors, publication year), study era, study country, study design, statistical analysis adjustment, study population], patient characteristics [age, sex, body surface area, body mass index, EuroScoreII, surgical approach (proportion of the minimally invasive approach), proportion of isolated AVR], and outcomes (primary outcomes: early mortality, transvalvular pressure gradients; secondary outcomes: aortic cross-clamp time, cardiopulmonary bypass time, paravalvular leak, pacemaker implantation, stroke, ICU stay). Data extraction was performed by two investigators (CW and YX), and discrepancies were resolved by a third investigator (JH).

### Quality assessment

We assessed the overall study quality using NEWCASTLE-OTTAWA SCALE (NOS) for observational studies (13), based on the three domains: selection of participators, comparability between study groups, and outcomes. Each study in this rating system (with a maximum of 9 stars) can receive up to 1 star for each numbered entry in the Selection and Outcome categories and up to 2 stars for the majority of entries in the Comparability category. A score of 9 stars received in the study indicates a low risk of bias, and a study that receives 8 or 7 stars is assessed as having a moderate risk of bias. In contrast, an assigned score of 6 or less indicates a high risk of bias.

### Outcomes

The primary outcomes of interest in the study were early mortality and transvalvular pressure gradients of the aortic valve after AVR. Early mortality was defined as 30-day all-cause mortality and in-hospital mortality. Transvalvular pressure gradients included mean transvalvular pressure gradients and peak transvalvular pressure gradients. The secondary outcomes of interest included ACC and CPB time, paravalvular leak (any paravalvular leak, moderate-to-severe paravalvular leak) after AVR, the need for pacemaker implantation, postoperative neurological events (stroke), and ICU stay.

### Statistical analysis

For continuous outcomes (transvalvular pressure gradients, aortic cross-clamp time, cardiopulmonary bypass time, and ICU stay), results were presented as the mean difference (MD) with a 95% confidence interval (CI) using an Inverse Variance fixed effect model, followed by real events, significance for effect estimate (*p*-value), *I*^2^ statistic, and *Q* statistic. We estimated the mean values and standard deviations using the formula if studies reported only the median and interquartile/overall range (14). The results of dichotomous outcomes (early mortality, paravalvular leak, pacemaker implantation, and stroke) were presented as the odds ratio (OR) with a 95% confidence interval (CI) using the Mantel–Haenszel fixed effect model. Total events, significance for effect estimate (*p*-value), *I*^2^ statistic, and *Q* statistic were also presented in pooling. When a moderate-to-high heterogeneity was discovered in the trial, the random effects model with the Inverse Variance or Mantel–Haenszel method was used in continuous or dichotomous outcomes, respectively. Operative time, including the aortic cross-clamp time and cardiopulmonary time, were pooled and presented in minutes, whereas ICU stay was presented in days. The magnitude of the statistical heterogeneity between studies was assessed using the Higgins *I*^2^ test, with rates of 25%, 50%, and 75% being indicative of low, moderate, and high heterogeneity, respectively (15). Furthermore, Cochran's *Q* statistic was used to assess the heterogeneity between the studies. We performed the leave-one-out sensitivity analysis to explore potential sources of heterogeneity by removing individual studies each time. Subgroup analysis was also performed to further stratify outcomes. We visually assessed potential publication bias by considering the asymmetry in the funnel plots of the effect size of each estimate against the standard error. A formal calculation of the possibility of publication bias was done by using Egger's test, which defines publication bias as significant if *p* < 0.1 (16). All study analyses were performed using Stata 16.0 (StataCorp LLC) and Review Manager Version 5.4.1 (The Cochrane Collaboration).

## Results

### Study search

**Figure 1:**
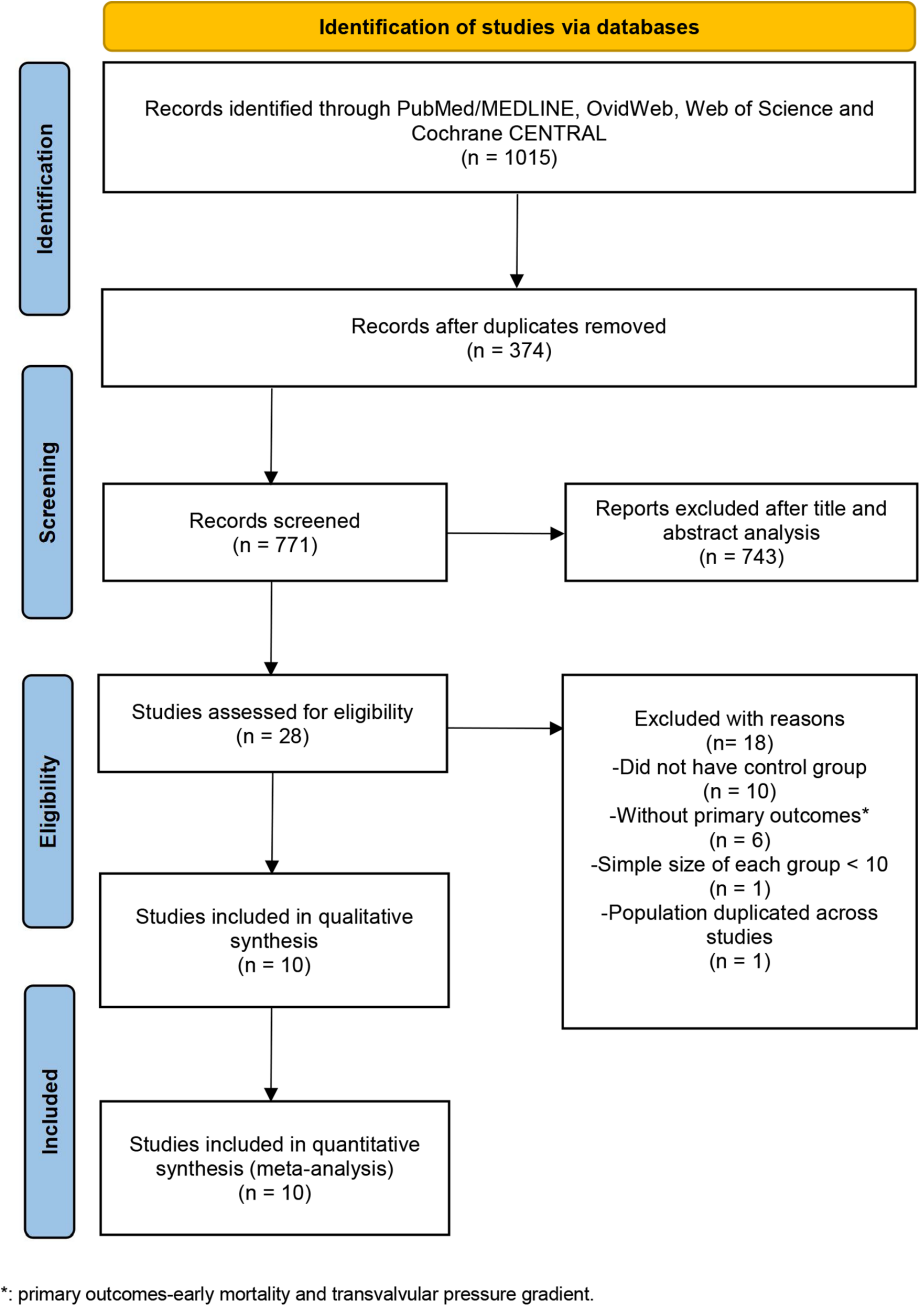
Preferred reporting items for systematic reviews and meta-analyses (PRISMA) flow diagram.

Our initial systematic electronic literature yielded 1,015 articles. After removing 374 duplicates, 771 articles were screened at the title/abstract level. Among these articles, 743 publications were excluded, which did not fulfill the selection criteria based on the title and abstract. With 28 articles remaining and assessed for eligibility, 10 publications were deemed eligible and included in the meta-analysis (Figure 1) (11, 17–25).

### Study characteristics and patient populations

The included 10 studies, nine full-text studies and one abstract with integral statistical reports, were all non-randomized studies (NRSs). Because there were three studies from the same registry, another two were used only to report supplementary data (19, 21). All studies covered 3,526 patients (sutureless group = 1,772 and rapid-deployment group = 1,754). Among these studies, propensity score matching was used in five studies (11, 17, 20, 24, 25), whereas in one study, the multivariable analysis method was used for determining early mortality in risk factor analysis (Table 1) (21). A larger proportion of male patients were enrolled in the rapid-deployment group. The mean age of patients in all studies ranged from 70 to 83 years, with most of them in their 70s (Table 2). Six studies reported about the body surface area in each group, with the rapid-deployment group having a statistically significant higher index (11, 18, 21, 22, 24, 25). One study reported data by dividing isolated AVR patients and combined AVR patients into two separate cohorts, which led us to perform a statistical analysis of these cohorts (25). All studies provided data on early mortality or transvalvular pressure gradients as primary outcomes, whereas specific secondary outcomes were unavailable in every study.

**Table 1:** Study characteristics.

Study (author, year)	Study era	Country	Study design	Statistical analysis adjustment	Study population		
					Total	SU (Perceval)	RD (Intuity)
Paolo Berretta et al., 2022 (isolated SURD-AVR)^a^	2007–2019	Multinational^b^	NRS	PSM	1,646	823	823
Paolo Berretta et al., 2022 (combined SURD-AVR)^a^	2007–2019	Multinational^b^	NRS	PSM	934	467	467
Liakopoulos et al., 2021	2012–2019	Germany	NRS	PSM	214	107	107
Martin Hartrumpf et al., 2020	2012–2017	Germany	NRS	None	119	80	39
Max Gotzmann et al., 2020	2016–2017	Germany	NRS	None	54	21	33
Augusto D’Onofrio et al., 2020	2011–2017	Italy	NRS	PSM	234	117	117
Paolo Berretta et al., 2019^c^	2007–2018	Multinational^b^	NRS	MVA^d^	1,418	1,011	407
Di Eusanio et al., 2018^c^	2007–2017	Multinational^b^	NRS	None	3,218	2,461	757
Stephan Ensminger et al., 2018	2011–2015	Germany	NRS	PSM	204	102	102
Federica Jiritano et al., 2016	2013–2015	Italy	NRS	None	43	16	27
Nguyen et al., 2015	2011–2015	Canada	NRS	PSM	78	39	39

SURD-AVR, sutureless and rapid-deployment aortic valve replacement; SU, sutureless; RD, rapid-deployment; NRS, non-randomized study; PSM, propensity score matching; MVA, multivariable analysis.

^a^ According to the studies, two sets of data were reported.

^b^ From Sutureless and Rapid Deployment Aortic Valve Replacement International Registry (SURD-IR): Australia, Austria, Belgium, Canada, France, Germany, Italy, and Switzerland.

^c^ Because this study was from the same registry as the study by Berretta et al. in 2021, it was only used to report data pertaining to pressure gradients, cardiopulmonary bypass time, and aortic cross-clamp time of patients overall, which were not reported in the study by Berretta et al. in 2021.

^d^ The MVA was performed in risk factor analysis for determining early mortality.

**Table 2:** Patient characteristics.

Study (author, year)	Age (year)		Male (%)		BSA (m^2^)		BMI (kg/m^2^)		EuroScore II (%)		Minimally invasive approach (%)		Isolated AVR (%)	
	SU (Perceval)	RD (Intuity)	SU (Perceval)	RD (Intuity)	SU (Perceval)	RD (Intuity)	SU (Perceval)	RD (Intuity)	SU (Perceval)	RD (Intuity)	SU (Perceval)	RD (Intuity)	SU (Perceval)	RD (Intuity)
Paolo Berretta et al., 2022 (isolated SURD-AVR)	75.4 ± 7.3	74.8 ± 7.3	42.5	45.3	1.81 ± 0.17	1.84 ± 0.19	27.6 ± 4.7	27.9 ± 4.9	7.8 ± 4.6^a^	7.1 ± 5.3^a^	74.6	80.1	63.8	63.8
Paolo Berretta et al., 2022 (combined SURD-AVR)	75.8 ± 7.2	75.4 ± 6.2	52.9	54	1.82 ± 0.18	1.85 ± 0.19	27.5 ± 4.4	27.4 ± 4.8	9.9 ± 6.7^a^	9.3 ± 7.1^a^	6.9	5.3	63.8	63.8
Oliver J. Liakopoulose et al., 2021	74.0 ± 8.0	76.0 ± 5.0	38	47	1.9 ± 0.2	1.9 ± 0.2	28 ± 5	28 ± 5	4 ± 5	4 ± 3	28	5.6	42	40
Martin Hartrumpf et al, 2020	72.3 ± 6.5	72.8 ± 5.5	53.8	56.4	−	−	28.85 ± 4.80	28.99 ± 5.30	6.44 ± 3.89^a^	11.78 ± 11.32^a^	−	−	75	48.7
Max Gotzmann et al., 2020	75.5 ± 6.6	71.7 ± 7.9	61.9	81.8	1.90 ± 0.15	1.94 ± 0.21	28.14 ± 4.55	27.85 ± 4.62	4.52 ± 4.44	5.21 ± 5.56	−	−	47.6	0
Augusto D’Onofrio et al., 2020	78.33 ± 6.70	77.97 ± 5.37	38.5	39.3	1.75 ± 0.19^b^	1.78 ± 0.18^b^	27.5 ± 4.9^b^	26.5 ± 4.2^b^	3.98 ± 3.06	3.95 ± 2.98	27.4	20.5	51.3	46.2
Paolo Berretta et al., 2019	76.7 ± 6.5	73.8 ± 7.8	32.6	46.9	1.83 ± 0.2	1.86 ± 0.2	27.5 ± 5	27.4 ± 5.2	9.4 ± 6.5^a^	6.8 ± 4.9^a^	46.6	80.8	100	100
Di Eusanio et al., 2018	76.8 ± 6.7	41.1	−	−	27.4 ± 4.8	11.3 ± 9.7	45.8	70.7						
Stephan Ensminger et al., 2018	74. 649 ± 5.2	41.1	−	−	27.4 ± 5.3	27.7 ± 4.5	2.2 ± 1.3^a^	33.3	30.4	68.6	71.6			
Federica Jiritano et al., 2016	75.94 ± 7.07	73.37 ± 6.79	62.5	66.7	1.77 ± 0.19	1.88 ± 0.24	−	−	9.26 ± 4.49	11.63 ± 4.15	−	−	100	100
Nguyen et al., 2015	83 ± 2	70 ± 7.6	−	−	−	−	−	−	4.7 ± 4.2	2.5 ± 1.8	−	−	−	−

AVR, aortic valve replacement; SU, sutureless; RD, rapid-deployment; BSA, body surface area; BMI, body mass index.

Values are presented as mean ± standard deviation.

^a^ Logistic EuroScore.

^b^ Data before propensity-score matching (PSM).

### Quality assessment

The methodological quality of each study varied, and the mean scores of the studies were 6.90 (SD = 0.99) out of 9 according to the Newcastle–Ottawa Scale (NOS), representing the included studies as moderate-to-high quality. A detailed quality assessment is presented in Supplementary Table S2.

### Early mortality

**Figure 2:**
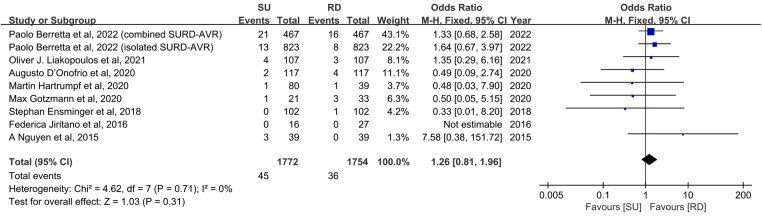
Odds ratio (OR) of early mortality in sutureless (SU) versus rapid-deployment (RD) aortic valve replacement. Overall pooled analysis from patients is shown. Compared with the RD group, the SU group is not associated with a significantly higher risk of early mortality (OR: 1.26; 95% CI: 0.81–1.96; *p* **=** 0.31; *I*^2 ^**= **0%). M–H, Mantel–Haenszel; CI, confidence interval.

All included studies reported early mortality, defined as 30-day all-cause mortality in five studies (11, 17, 18, 22, 24) and in-hospital mortality in another three studies (20, 23, 25), respectively. Effect sizes were expressed by ORs, whereas ORs were not calculated in one study because the early mortality in both groups was 0 (18). The calculated overall early mortality rate was 2.3%, being 2.5% in patients receiving Perceval valve implantation and 2.1% in those who underwent Intuity valve implantation (*p* = 0.31). The SU group showed no statistically significant difference in early mortality rates compared with the RD group (8 studies and 3,526 patients, OR: 1.26; 95% CI: 0.81–1.96; *p* **=** 0.31; *I*^2 ^**= **0%, Figure 2). No significant publication bias was observed, which was assessed by considering the asymmetry in the funnel plot visually and formally by using Egger's regression test (*p* = 0.5190, Supplementary Figure S5A). Finally, a sensitivity analysis was used to examine the influence of each study on the OR by excluding one individual study at one time. The exclusion of each study did not significantly change the pooled OR, and the estimates for each case were within the overall 95% confidence interval.

### Transvalvular pressure gradients

#### Mean transvalvular pressure gradients

**Figure 3:**
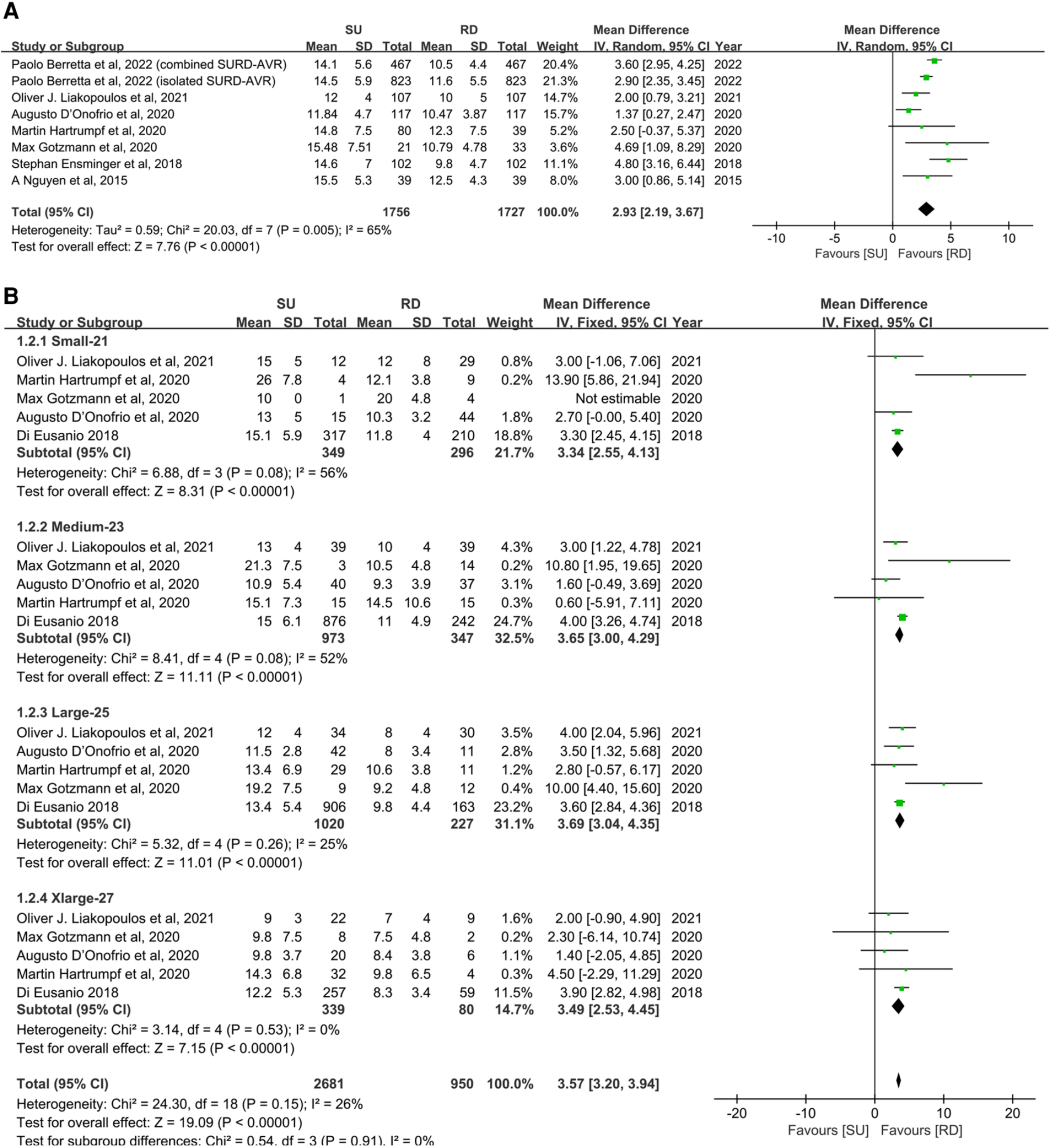
Mean difference (MD) of mean transvalvular pressure gradients (mmHg) in sutureless (SU) versus rapid-deployment (RD) aortic valve replacement. Overall pooled analyses from patients (**A**), subgroup 1 (**B**) and subgroup 2 (**C**) are shown. Subgroup 1 matches SU with RD valve sizes as small with 21 mm, medium with 23 mm, large with 25 mm, and extralarge with 27 mm, and subgroup 2 matches SU with RD valve sizes as small with 19 mm, medium with 21 mm, large with 23 mm, and extralarge with 25 mm. Compared with the RD group, the SU group is associated with a significantly higher mean transvalvular pressure gradient in patients overall (MD: 2.93; 95% CI: 2.19–3.67; *p* < 0.00001; *I*^2 ^=** **65%), subgroup 1 (MD: 3.57; 95% CI: 3.20–3.94; *p* **<** 0.00001; *I*^2 ^=** **26%) and subgroup 2 (MD: 1.68; 95% CI: 0.77–2.58; *p* **=** 0.0003; *I*^2 ^**= **74%). SD, standard deviation; IV, inverse-variance; CI, confidence interval.

Overall, the patients' mean transvalvular pressure gradients were presented in seven studies (11, 17, 20, 22–25), and five studies reported the mean transvalvular pressure gradients in each size of both valve types (11, 19, 22–24). The pooled analysis from seven studies covering 3,483 patients demonstrated that the SU group was associated with statistically significant higher mean transvalvular pressure gradients in patients overall, compared with the RD group (MD: 2.93; 95% CI: 2.19–3.67; *p* < 0.00001; *I*^2 ^**= **65%, Figure 3A). Next, we performed subgroup analyses by matching the sizes of the Perceval and Intuity valves to further explore the relationship between valve size and transvalvular pressure gradients and make a hierarchical contrast between the two types of valves. Subgroup 1 compared SU with RD valve sizes under radical matching by small with 21 mm, medium with 23 mm, large with 25 mm, and extralarge with 27 mm, whereas subgroup 2 compared SU with RD valve sizes under conservative matching by small with 19 mm, medium with 21 mm, large with 23 mm, and extralarge with 25 mm. Subgroup analyses demonstrated that under radical matching of valve size, the SU group was still associated with statistically significant higher mean transvalvular pressure gradients in each size-matching compared with the RD group (MD: 3.57; 95% CI: 3.20–3.94; *p* **<** 0.00001; *I*^2 ^**= **26%, Figure 3B). However, under conservative matching of valve size, it presented a lower mean transvalvular pressure gradient in the S SU valve than the 19 mm RD valve, but it was still significantly higher in the M, L, and XL SU valves than in the 21, 23, and 25 mm RD valves, respectively (MD: 1.68; 95% CI: 0.77–2.58; *p* **=** 0.0003; *I*^2 ^**= **74%, Figure 3C). No significant publication bias was observed in patients overall, which was assessed by considering the asymmetry in the funnel plot visually and formally by using Egger's regression test (*p* = 0.5879, Supplementary Figure S5B).

**Figure:**
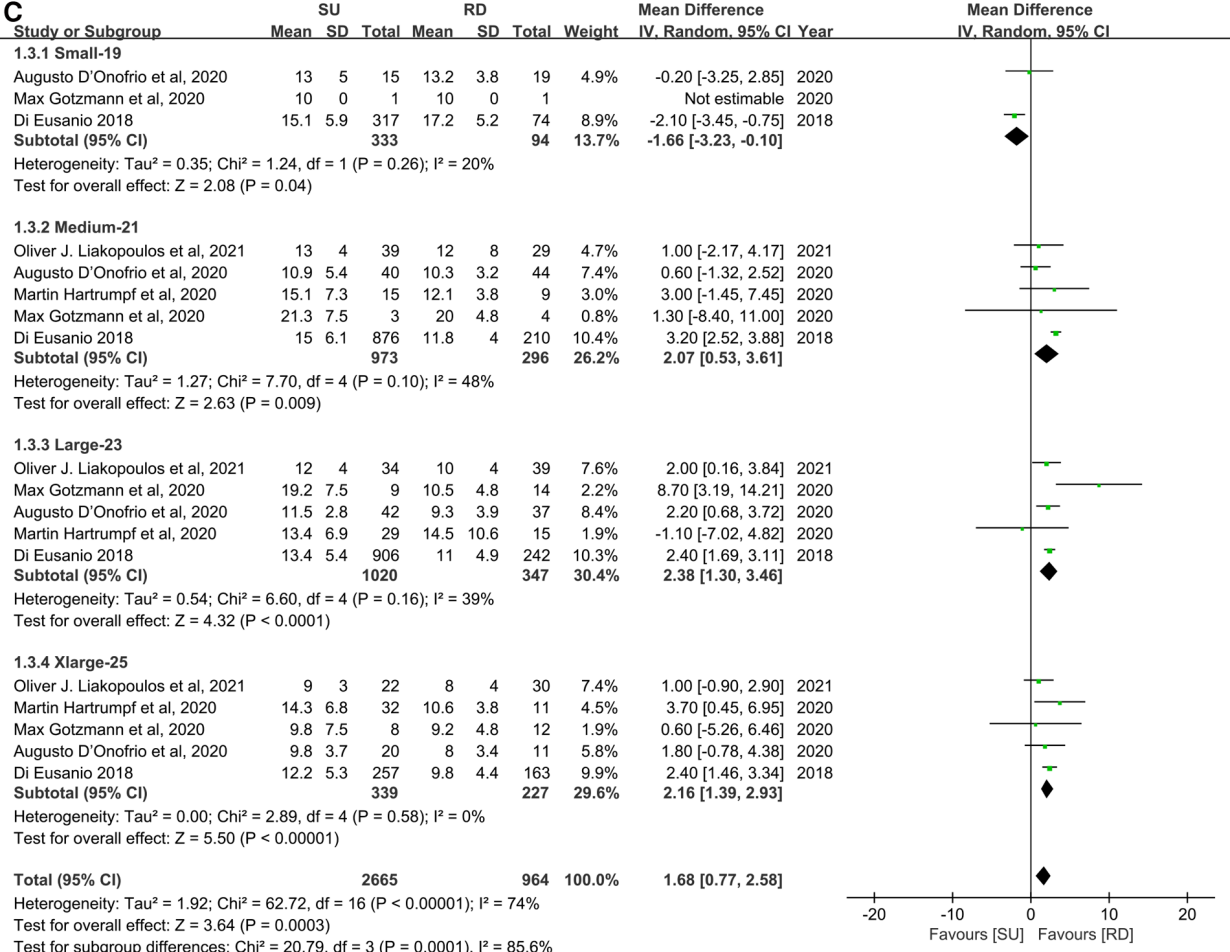


#### Peak transvalvular pressure gradients

**Figure 4:**
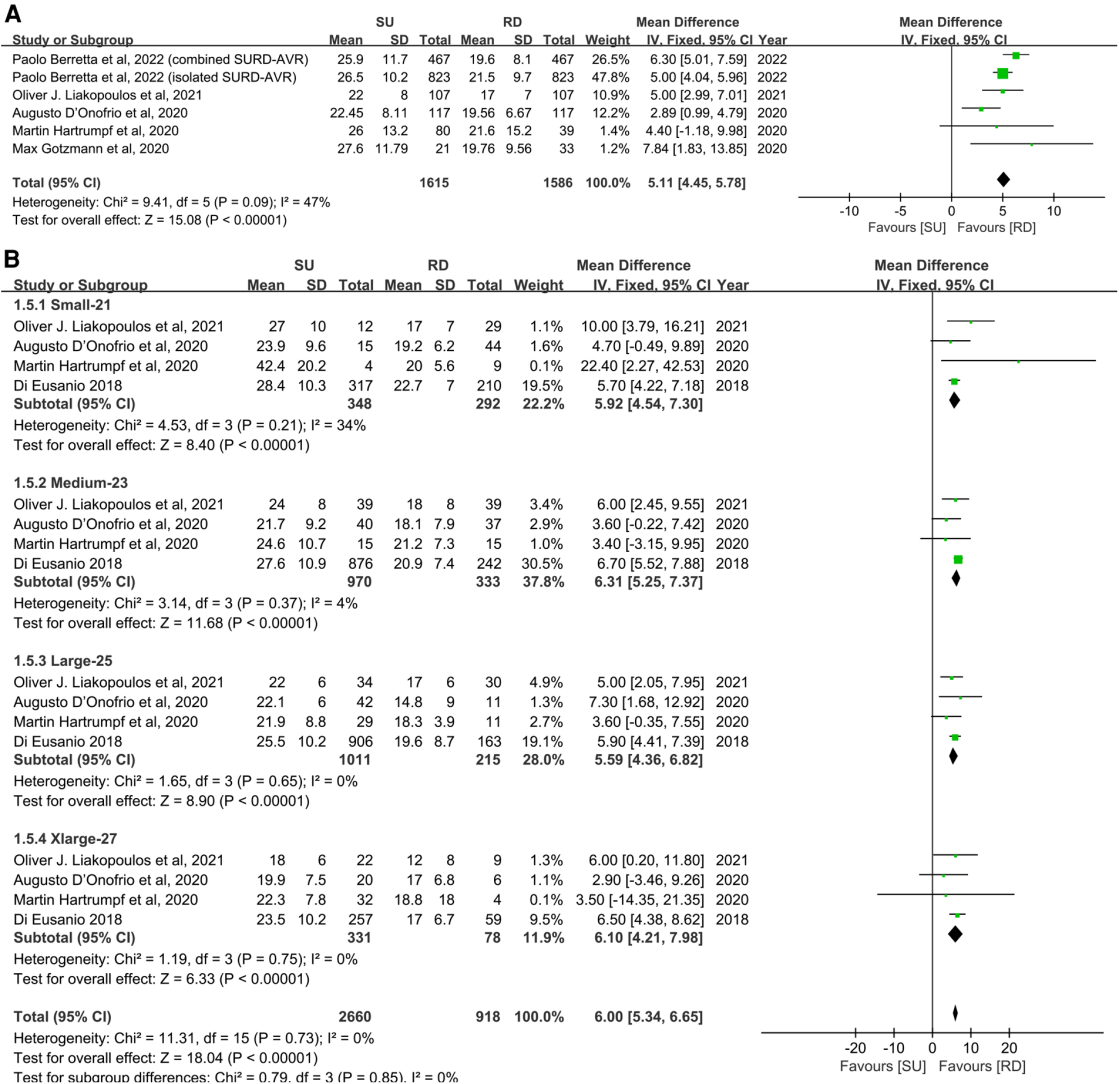
Mean difference (MD) of peak transvalvular pressure gradients (mmHg) in sutureless (SU) versus rapid-deployment (RD) aortic valve replacement. Overall pooled analyses from patients (**A**), subgroup 1 (**B**), and subgroup 2 (**C**) are shown. Subgroup 1 matches SU with RD valve sizes as small with 21 mm, medium with 23 mm, large with 25 mm, and extralarge with 27 mm. Subgroup 2 matches SU with RD valve sizes as small with 19 mm, medium with 21 mm, large with 23 mm, and extralarge with 25 mm. Compared with the RD group, the SU group is associated with significantly higher peak transvalvular pressure gradients in patients overall (MD: 5.11; 95% CI: 4.45–5.78; *p* < 0.00001; *I*^2 ^**= **47%), subgroup 1 (MD: 6.00; 95% CI: 5.34–6.65; *p* **<** 0.00001; *I*^2 ^**= **0%) and subgroup 2 (MD: 2.86; 95% CI: 1.18–4.55; *p* **=** 0.0008; *I*^2 ^**= **82%). SD, standard deviation; IV, inverse-variance; CI, confidence interval.

For peak transvalvular pressure gradients, statistical analyses demonstrated the same tendency as the mean transvalvular pressure gradients. Five studies and 3,201 patients were covered in an overall pooled analysis (11, 22–25), which demonstrated that the SU group was associated with statistically significant higher peak transvalvular pressure gradients in patients overall, compared with the RD group (MD: 5.11; 95% CI: 4.45–5.78; *p* < 0.00001; *I*^2 ^**= **47%, Figure 4A). Subgroup analyses were also performed by small with 21 mm, medium with 23 mm, large with 25 mm, and extralarge with 27 mm as radical matching and small with 19 mm, medium with 21 mm, large with 23 mm, and extralarge with 25 mm as conservative matching. For radical matching, the SU group was associated with statistically significant higher peak transvalvular pressure gradients in each size-matching compared with the RD group (MD: 6.00; 95% CI: 5.34–6.65; *p* **<** 0.00001; *I*^2 ^**= **0%, Figure 4B). For conservative matching, the peak pressure gradients in the SU group were still significantly higher in the M, L, and XL SU valves than in the 21, 23, and 25 mm RD valves (MD: 2.86; 95% CI: 1.18–4.55; *p* **=** 0.0008; *I*^2 ^**= **82%, Figure 4C). No significant publication bias was observed in patients overall, which was assessed by taking into account the asymmetry in the funnel plot visually and formally by using Egger's regression test (*p* = 0.8425, Supplementary Figure S5C).

**Figure:**
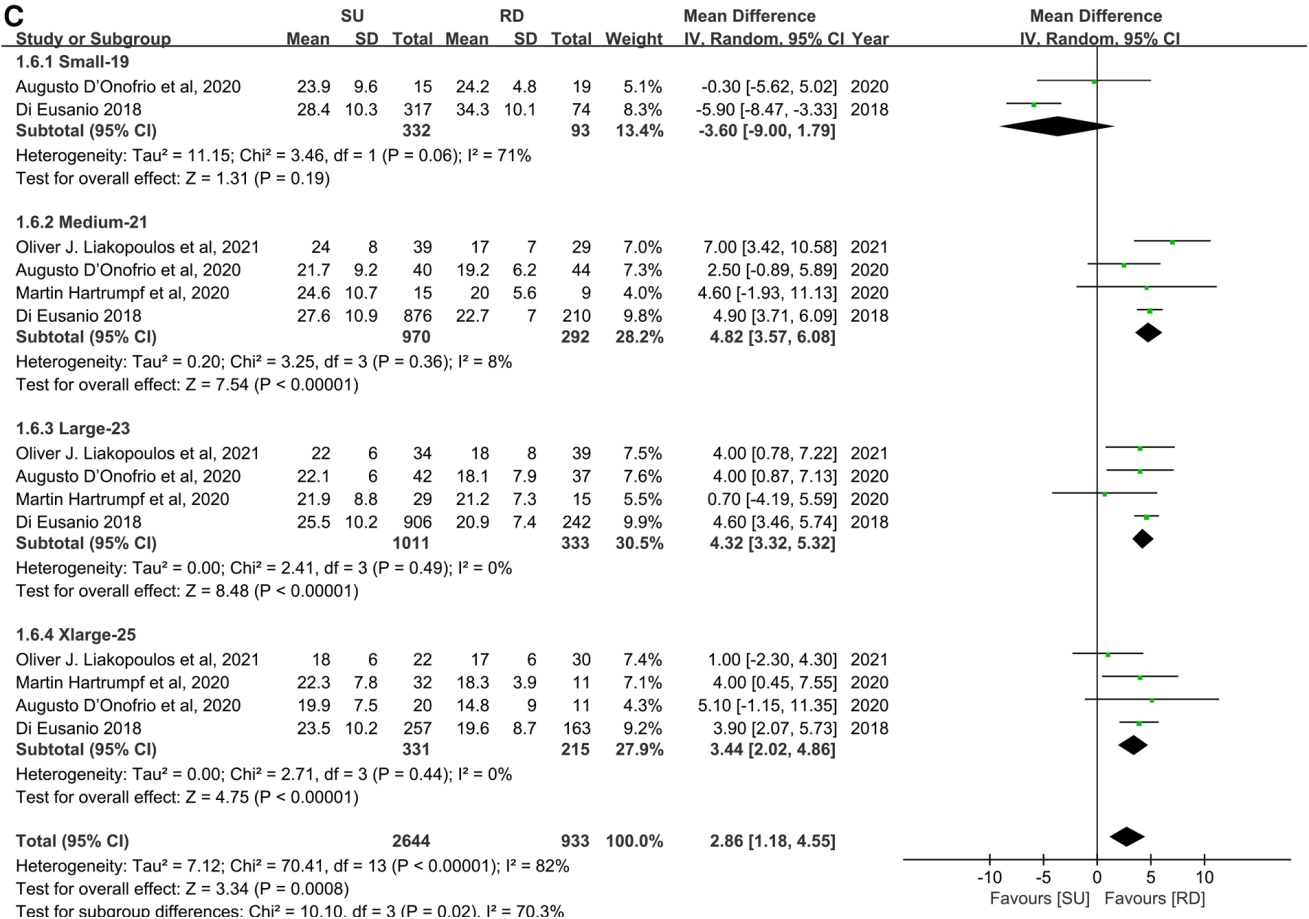


### Secondary outcomes

**Figure 5:**
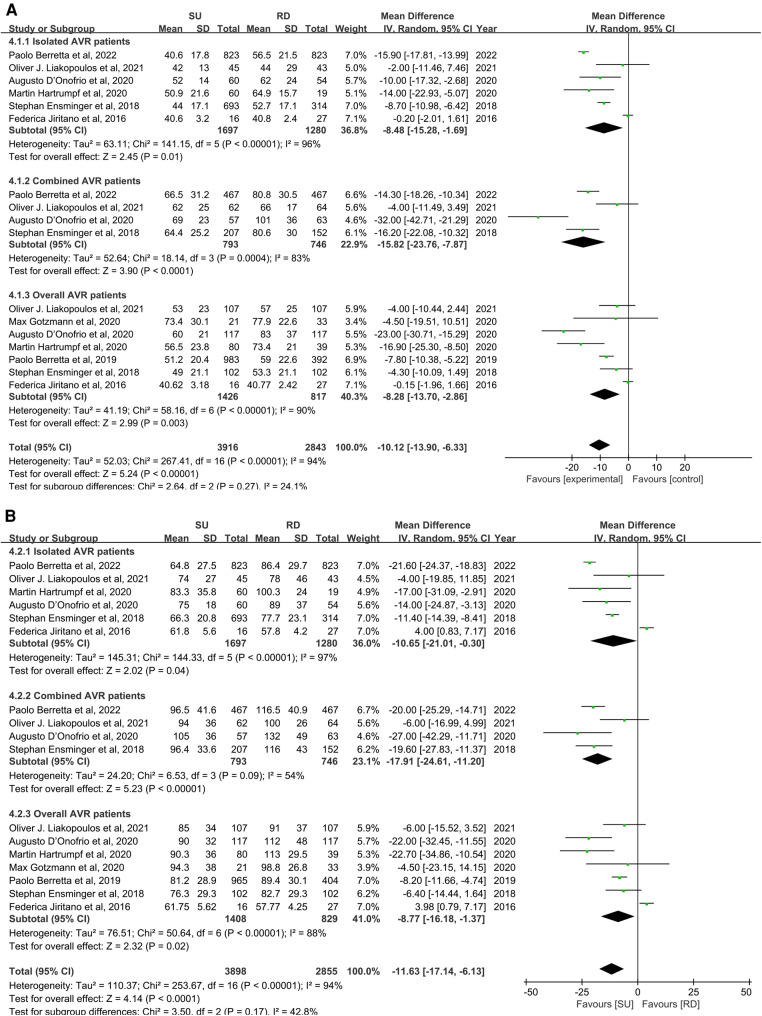
Mean difference (MD) of aortic cross-clamp (ACC) (**A**) and cardiopulmonary bypass (CPB) (**B**) times in sutureless (SU) versus rapid-deployment (RD) aortic valve replacement (AVR). Overall pooled analyses from isolated AVR patients, combined AVR patients, and AVR patients are shown. Compared with the RD group, the SU group is associated with a significantly less aortic cross-clamp time (MD: −10.12; 95% CI: −13.90 to −6.33; *p* **<** 0.00001; *I*^2 ^**= **94%), and similarly, with a significantly less cardiopulmonary bypass time (MD: −11.63; 95% CI: −17.14 to −6.13; *p* **<** 0.0001; *I*^2 ^=** **94%). SD, standard deviation; IV, inverse-variance; CI, confidence interval.

For secondary outcome studies, extracted estimates were reported in the supplementary material. Overall pooled analyses from isolated AVR patients, combined AVR patients, and AVR patients demonstrated that, compared with the RD group, the SU group was associated with a significantly less aortic cross-clamp time (MD: −10.12; 95% CI: −13.90 to −6.33; *p* < 0.00001; *I*^2^ = 94%, Figure 5A), and similarly, with a significantly less cardiopulmonary bypass time (MD: −11.63; 95% CI: −17.14 to −6.13; *p* **<** 0.0001; *I*^2 ^**= **94%, Figure 5B). There were no statistically significant differences between the SU group and the RD group for any paravalvular leak (OR: 1.95; 95% CI: 1.01–3.77; *p* **=** 0.05; *I*^2 ^**= **75%, Supplementary Figure S1A), paravalvular leak (moderate to severe) (OR: 1.07; 95% CI: 0.61–1.87; *p* **=** 0.82; *I*^2 ^**= **0%, Supplementary Figure S1B), pacemaker implantation (OR: 1.16; 95% CI: 0.92–1.47; *p* **=** 0.20; *I*^2 ^**= **0%, Supplementary Figure S2), stroke (OR: 1.07; 95% CI: 0.70–1.64; *p* **=** 0.75; *I*^2 ^**= **0%, Supplementary Figure S3), and intensive care unit (ICU) stay (MD: −0.03; 95%CI: −0.37 to 0.31; *p* **=** 0.87; *I*^2 ^**= **75%, Supplementary Figure S4). A visual assessment of the symmetry of the funnel plots suggested that there was no significant publication bias, and a formal assessment by using Egger's test confirmed this point (Supplementary Figure S5).

## Discussion

In this study, we conducted a meta-analysis covering 10 non-randomized trials and 3,526 patients, highlighting two key findings. First, compared with the RD group, the SU group was associated with statistically significant higher mean and peak transvalvular pressure gradients of the aortic valve. Second, the SU group was associated with an overall decrease of ACC and CPB times for 10.12 min and 11.63 min, respectively, compared with the RD group. In terms of early mortality, paravalvular leak, moderate-to-severe paravalvular leak, pacemaker implantation, stroke, or ICU stay, data analysis revealed commonalities between the two groups.

Our honest opinion is that selecting the appropriate valve for a defined patient based on the information revealed in our study remains a challenging proposition. Although our study revealed that the two valves displayed varied hemodynamic and intraoperative performances, this did not translate into different clinical outcomes for patients. However, there is still a lack of medium- to long-term follow-up and comprehensive data to determine critical outcomes in terms of survival and major adverse cardiac and cerebral events. Therefore, it is important to have risk predictors that impact the long-term prognosis for the two valves when analyzing the advantages and disadvantages of each valve, with implications to guide clinicians in their selection.

It has been proved that SURD-AVR possesses a better hemodynamic function compared with SAVR (25). The following interpretations, according to several investigations, could account for this satisfactory observation: (1) the non-pledged sutures may contribute to a huger laminar flow; (2) as the thin stent allows the leaflets to move freely without being firmly bound to bulky stents, the Perceval valve result in the pressure gradients drops; (3) seated below the annulus, the skirt frame of the stent of the Intuity valve has a flared configuration in the left ventricular outflow, which may play a role in active constriction limitation in the left ventricular outflow tract (LVOT) (25–29).

Our meta-analysis performed using both radical and conservative matching revealed that when compared with the Intuity valve, the Perceval valve had statistically significant higher mean transvalvular pressure gradients across all patients and subgroup analyses.

Theoretically, in terms of valve structure, as the Intuity valve has the valve annulus stent covered by a polyester sealing cloth (8, 30), the Perceval valve could offer a larger effective outflow orifice area, leading to its better hemodynamic performance. Nevertheless, this hypothesis is in stark contrast to our meta-analysis observation, which should be highlighted purposely. A previous study reported this theory-contradicted finding (31). If the stent in the Perceval valve undergoes compression or deformation after the prosthesis implantation procedure, it could indicate oversizing relative to the annulus or procedural misoperation by the surgeon, potentially resulting in a high gradient. This grossly oversized prosthesis mismatched with the patient tends to spring back, causing incomplete valve opening and contact loss from the annulus, which possibly results in high paravalvular leakage, besides an increase in the pressure gradients. Several published studies reported that Perceval valve rebounds were observed in clinical implantation and laboratories (32, 33). This feasible explanation for cracking the paradox of valve-pressure gradients is consistent with the trend of paravalvular leak in our meta-analysis results (SU group: 184 in 1,530; RD group: 96 in 1,542. OR: 1.95; 95% CI: 1.01–3.77; *p* **=** 0.05; *I*^2 ^**= **75%). Strikingly, another theoretical possibility was proposed by Campbell D. Flynn et al. to the effect that the Intuity valve that has better pressure gradients focuses on the valve skirt (34). The subannular balloon-expanded valve skirt in the Intuity valve is proposedly attributed to the recognized excellent transvalvular pressure gradients in the RD group, in which the LVOT is enlarged, promoting an increase in blood flow through the valve annulus (35, 36). Although the expandable frame skirt in the Intuity valve may enlarge the LVOT, it is certain that the stent located at the leaflet attachment margin narrows the orifice area. To sum up, our study was more inclined to conclude that the incomplete valve opening in the Perceval valve caused a higher gradient and showed a higher tendency toward paravalvular leak, for which further studies should confirm the potential mechanism.

Furthermore, it is necessary to highlight that the difference in valve gradient between these two groups (MD = 2.93 mmHg in mean aortic pressure gradients; MD = 5.11 mmHg in peak aortic pressure gradients) did not translate into differences in early clinical outcomes. In the meantime, the hemodynamic performance of the two valves needs to be further followed up and explored. Only then will it be possible to show the impact of the difference in transvalvular pressure gradients on the long-term prognosis of patients who received SU and RD-AVR. Notably, patients with smaller aortic annuli who undergo aortic valve replacement often exhibit higher transvalvular pressure gradients, and the presence of a small aortic annulus may augment the risk of patient–prosthesis mismatch (37, 38). Hence, it is plausible that the Intuity valve may offer superior postoperative benefits to patients with a small aortic annulus.

In our study, overall pooled analyses from isolated AVR patients, combined AVR patients, and AVR patients demonstrated that, compared with the RD group, the SU group was associated with significantly less aortic cross-clamp time (MD: −10.12; 95% CI: −13.90 to −6.33; *p* < 0.00001; *I*^2^ = 94%). We suspected that this discrepancy arose because of these two valves possessing distinct suture structures. The Perceval valve is a bovine pericardium prosthesis attached to the automated anchor used for stabilization and a fastened implantation site. When the valve is placed down to the annulus, three intercommissural sutures are used for guiding, which will be removed after valve deployment is completed (8, 39). In addition, the Perceval valve with a collapsed design may maximize visualization and simplify implantation (25). In contrast, three braided, non-pledged sutures are placed at the bottom of every valve sinus using a figure-of-eight or horizontal mattress technique without removal if the Intuity valve is selected for use in the AVR. Once annular seating is verified, the balloon will be inserted through the holder, and the stent will be deployed by inflating it to the appropriate level of pressure with saline for 10 s (40). Therefore, the Perceval valve is the only one that precisely matches the definition of “sutureless” during operation. Because of these structural and procedural differences with the Perceval valve, some opponents have argued that the Intuity valve cannot strictly be labeled as a “rapid-deployment” valve (30). However, it was noted that the magnitude assessment showed high heterogeneity, with subgroup analysis and leave-one-out sensitivity analysis being inefficient for elimination.

Postoperative mortality and morbidity are strongly associated with the duration of both ACC and CPB. A previously published study has indicated that ACC time is a critical and independent risk predictor of severe cardiovascular morbidities, with the risk increasing by 1.4% for each additional minute of ACC time (41). Kenji Lino et al. also revealed that ACC time serves as an independent risk predictor of postoperative morbidity for aortic valve replacement, with a prolonged ACC duration significantly increasing the rates of renal failure, gastrointestinal complications, pneumonia, and multiorgan failure (42). In addition, a study conducted in China has reported that CPB time is independently linked to an increased risk of acute kidney injury following surgery for acute DeBakey Type I aortic dissection (43). Therefore, for high-risk patients undergoing AVR, reducing the ACC and CPB times may confer substantial advantages in using the Perceval valve, particularly for patients with pre-existing organ damage and infections or for those undergoing redo surgery (44, 45).

Two meta-analyses (30, 34) anchored on the comparison of the sutureless and rapid-deployment aortic valves in SURD-AVR had been published before our study was done. Nevertheless, two aspects (paravalvular leak and pacemaker implantation) of our analysis presented negative results, showing slight differences with the conclusions of the two previous studies. Published studies may be responsible for causing discrepancies at different times, discrepancies in inclusion criteria, and differences in the exact definition of study outcomes. However, it is noteworthy and distinctive that compared with other studies to date, our study covers the largest period, the largest number of patients, the most significant number of included studies, all types of early clinical results, and the use of two valve size gradient matching methods, to enable a comprehensive and objective comparative analysis.

There are several limitations in our analysis that merit a scrupulous consideration. First, we included only 10 studies overall; also, we did not include any RCT. Although propensity score matching was performed in more than half of the included studies to equalize confounders in non-randomized studies similar to randomization, there is no denying the potential selection bias of our investigators. Second, SURD-IR and Germany are the majority contributors to the patient data source that we collected in the study, which means a more homogeneous region and race limit the generalizability of analysis results. Third, because follow-up was patchy across studies, there is a need for comparing the efficacy and durability of the two valves in the medium and long term. Fourth, the results of ACC and CPB times showed high heterogeneity. Even though we performed leave-one-out sensitivity analysis and subgroup analysis, we still could not well locate and reduce the source of heterogeneity. Fifth, although we performed subgroup analysis by valve size to ensure precise matching, no clear distinction could be perceived between Perceval S (Livanova PLC, London, UK) and Perceval S (Sorin Group, Saluggia, Italy) in the results of pooled estimates reported in our study. Last, potential publication bias cannot be definitively ruled out, even though both Egger's test and the funnel plots suggest no potential publication bias.

## Conclusion

Although further trials and reviews are required for making a more detailed and deterministic comparison between the valves in SURD-AVR, particularly clinical outcomes in the medium and long term in practice, our findings lend support to the notion that sutureless aortic valve replacement is associated with significantly higher postoperative mean and peak transvalvular pressure gradients of the aortic valve compared with rapid-deployment aortic valve replacement in overall and subgroup analyses. Sutureless aortic valve replacement provided visible benefits to patients in terms of intraoperative performance as there was a significant reduction in ACC and CPB times compared with rapid-deployment aortic valve replacement. We also discussed the role of different risk predictors to guide valve selection. In conclusion, clinical decision-making should necessitate thoughtful valve selection for all patients prior to SURD-AVR, and in this context, it can be said that both Perceval and Intuity valves are rising stars in the bioprosthesis firmament, complementing each other very well.

## Data Availability

The original contributions presented in the study are included in the article/**Supplementary Material**, further inquiries can be directed to the corresponding author.
