# Supplementary material for: Sutureless vs. rapid-deployment valve: a systemic review and meta-analysis for a direct comparison of intraoperative performance and clinical outcomes
Source: Front Cardiovasc Med. 2023 May 15;10:1123487. doi: 10.3389/fcvm.2023.1123487 (PMC10225698; doi:10.3389/fcvm.2023.1123487)
Supplement: Supplementary file 1 [file Datasheet1.docx]

Supplementary figures caption:
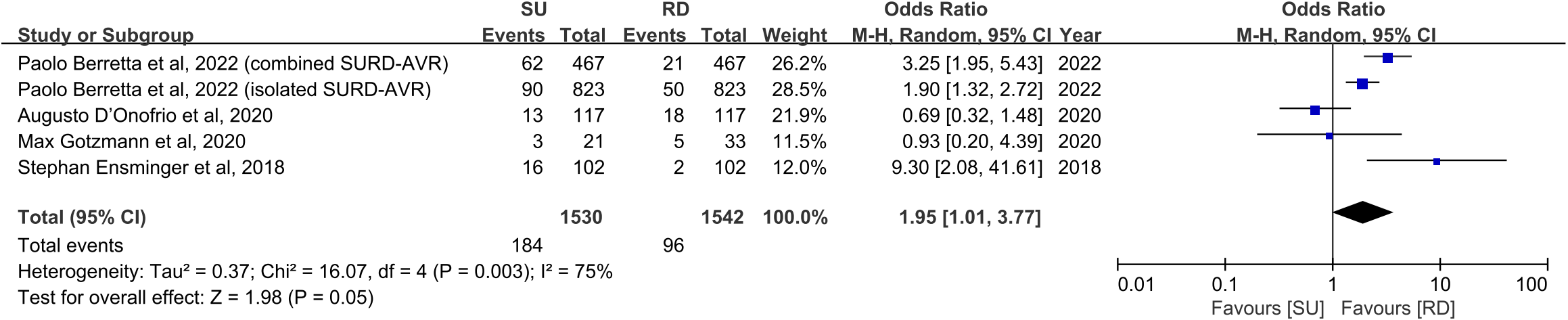
(A)


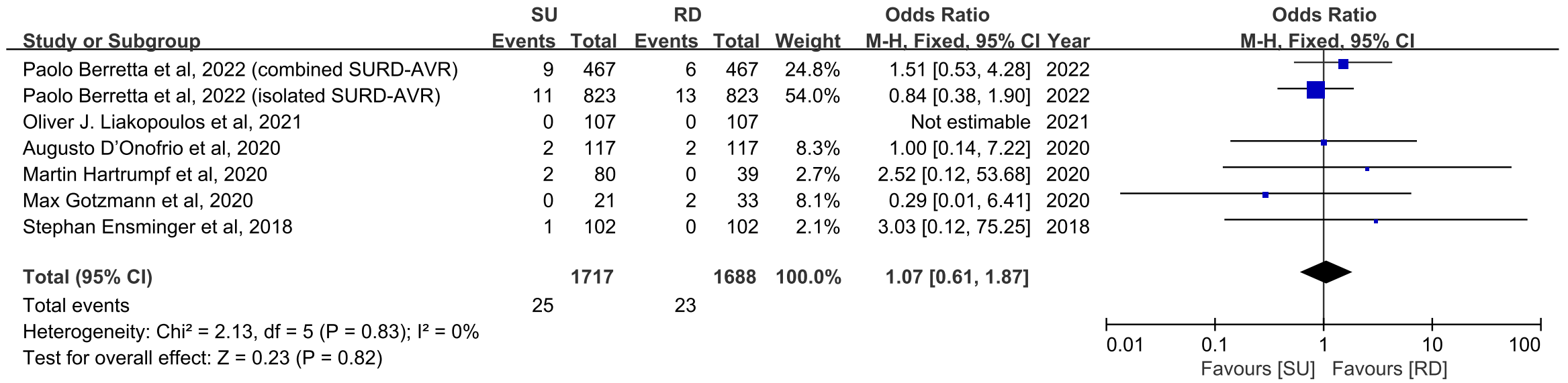
(B)

**Figure S1:** Odds ratio (OR) of any paravalvular leak (A) and paravalvular leak (moderate-severe) (B) in sutureless (SU) versus rapid-deployment (RD) aortic valve replacement. Compared with RD group, SU group was not associated with significantly higher risk of any paravalvular leak (OR: 1.95; 95%CI: 1.01 to 3.77; p **=** 0.05; I^2^ **=** 75%), whereas SU group showed no statistically significant difference of risk of paravalvular leak (moderate-severe) compared with RD group (OR: 1.07; 95%CI: 0.61 to 1.87; p **=** 0.82; I^2^ **=** 0%). M-H **=** Mantel-Haenszel, CI **=** confidence interval.

**
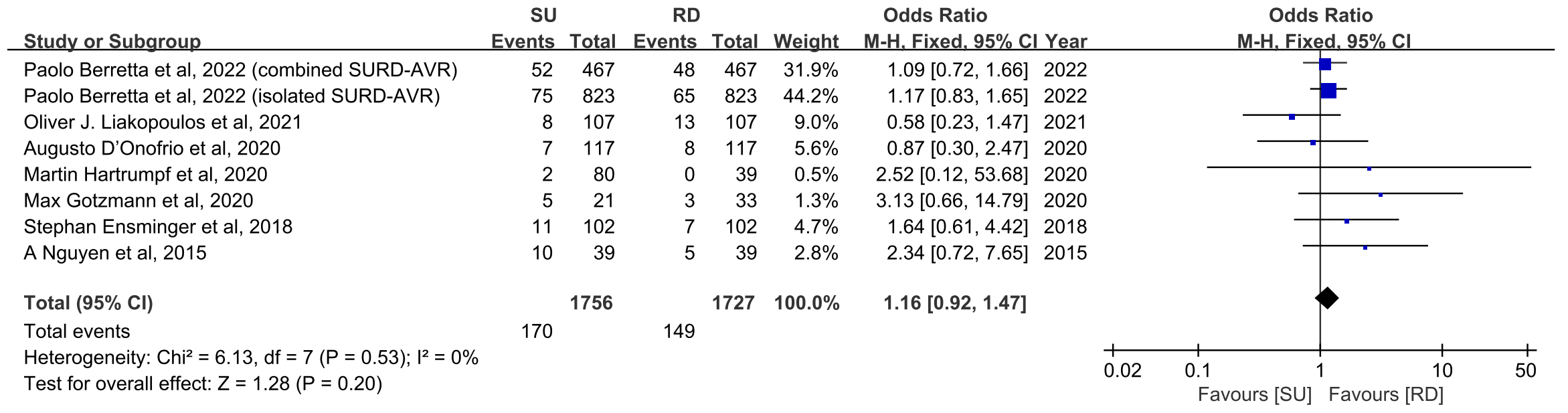
Figure S2:** Odds ratio (OR) of pacemaker implantation in sutureless (SU) versus rapid-deployment (RD) aortic valve replacement. Compared with RD group, SU group was not associated with significantly higher risk of pacemaker implantation (OR: 1.16; 95%CI: 0.92 to 1.47; p **=** 0.20; I^2^ **=** 0%). M-H **=** Mantel-Haenszel, CI **=** confidence interval.

**
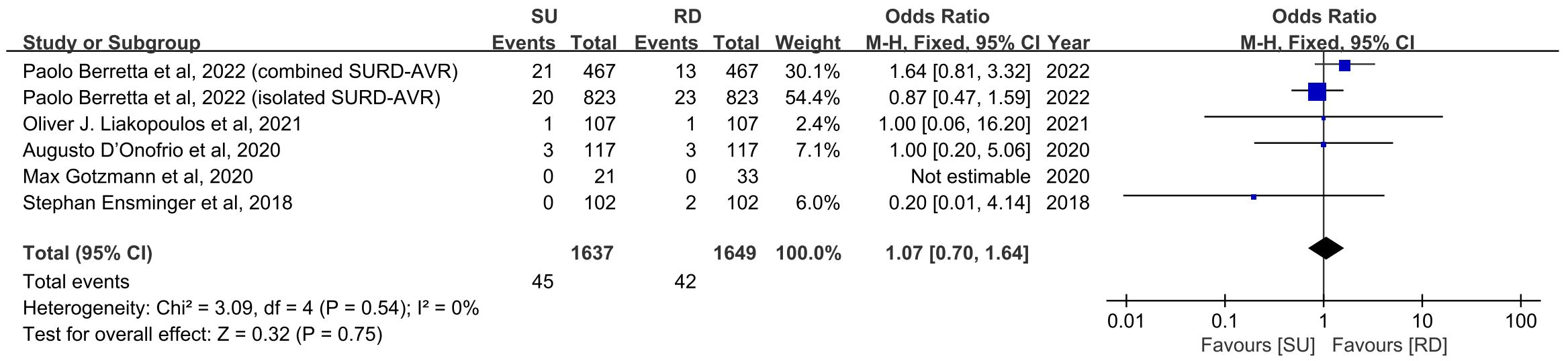
Figure S3:** Odds ratio (OR) of stroke in sutureless (SU) versus rapid-deployment (RD) aortic valve replacement. SU group showed no significantly difference of risk of stroke compared with RD group (OR: 1.07; 95%CI: 0.70 to 1.64; p **=** 0.75; I^2^ **=** 0%). M-H **=** Mantel-Haenszel, CI **=** confidence interval.

**
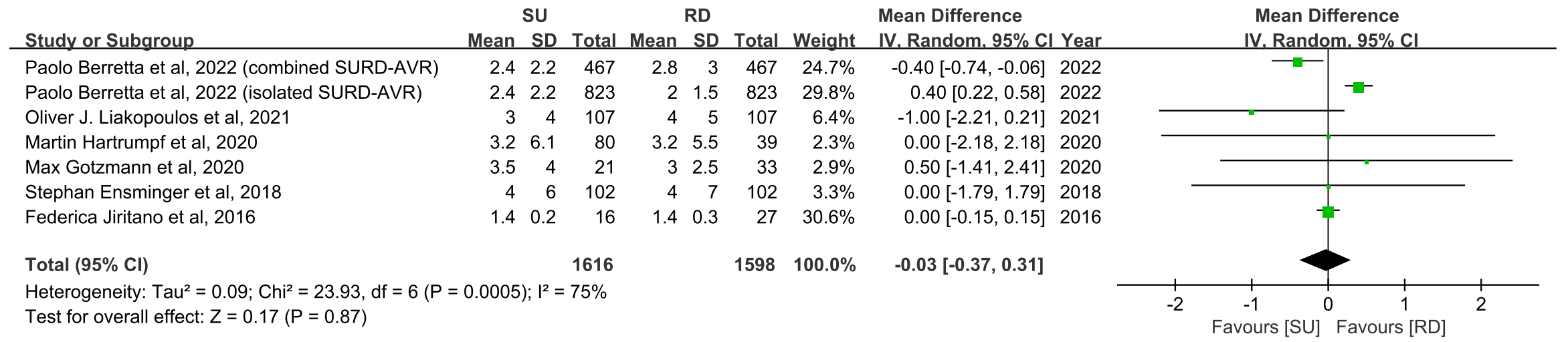
Figure S4:** Mean difference (MD) of intensive care unit (ICU) stay in sutureless (SU) versus rapid-deployment (RD) aortic valve replacement. SU group showed no significantly difference of ICU stay compared with RD group (MD: -0.03; 95%CI: -0.37 to 0.31; p **=** 0.87; I^2^ **=** 75%). SD **=** standard deviation, IV **=** Inverse-Variance, CI **=** confidence interval.


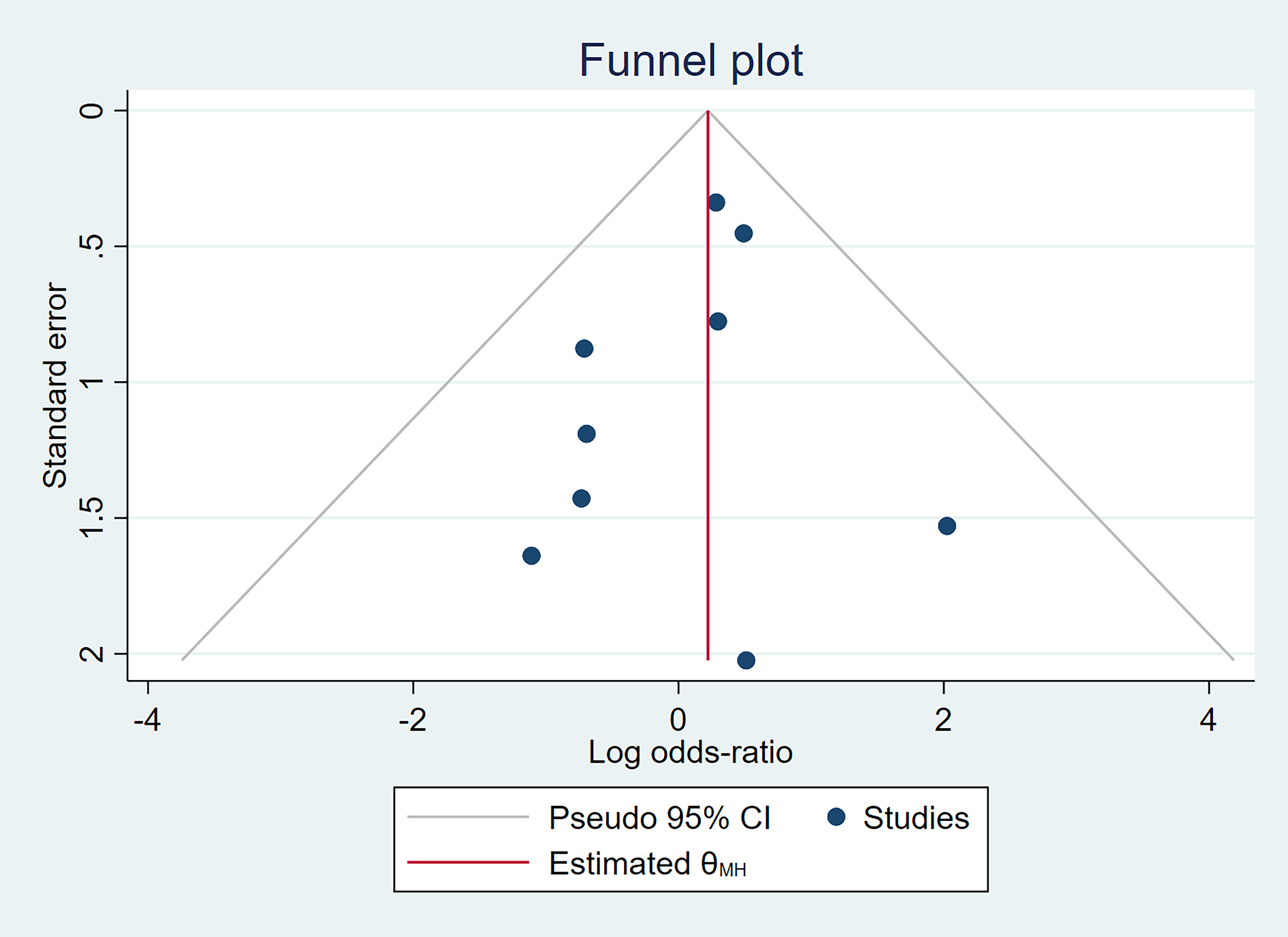
(A)
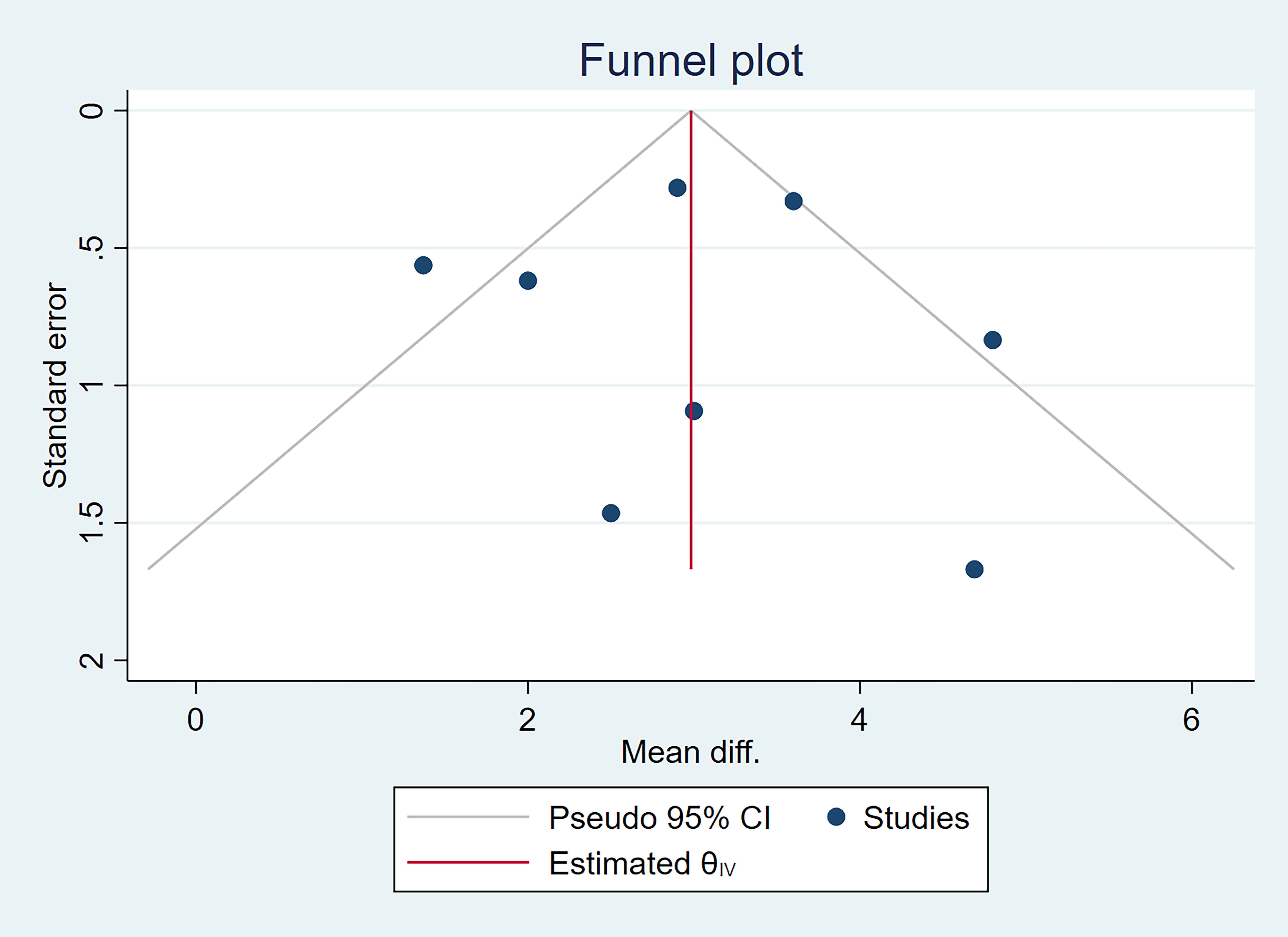
(B)
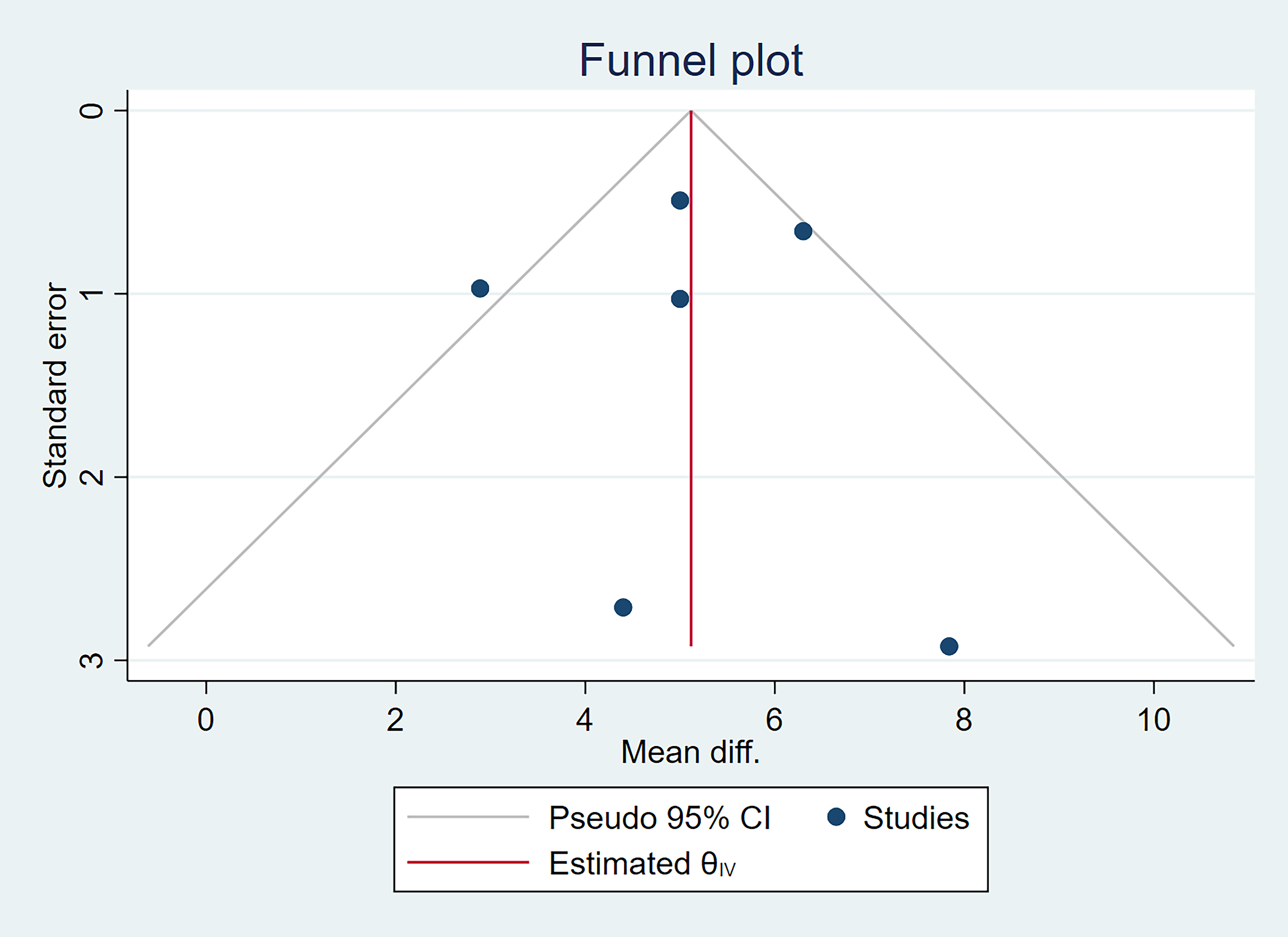
(C)
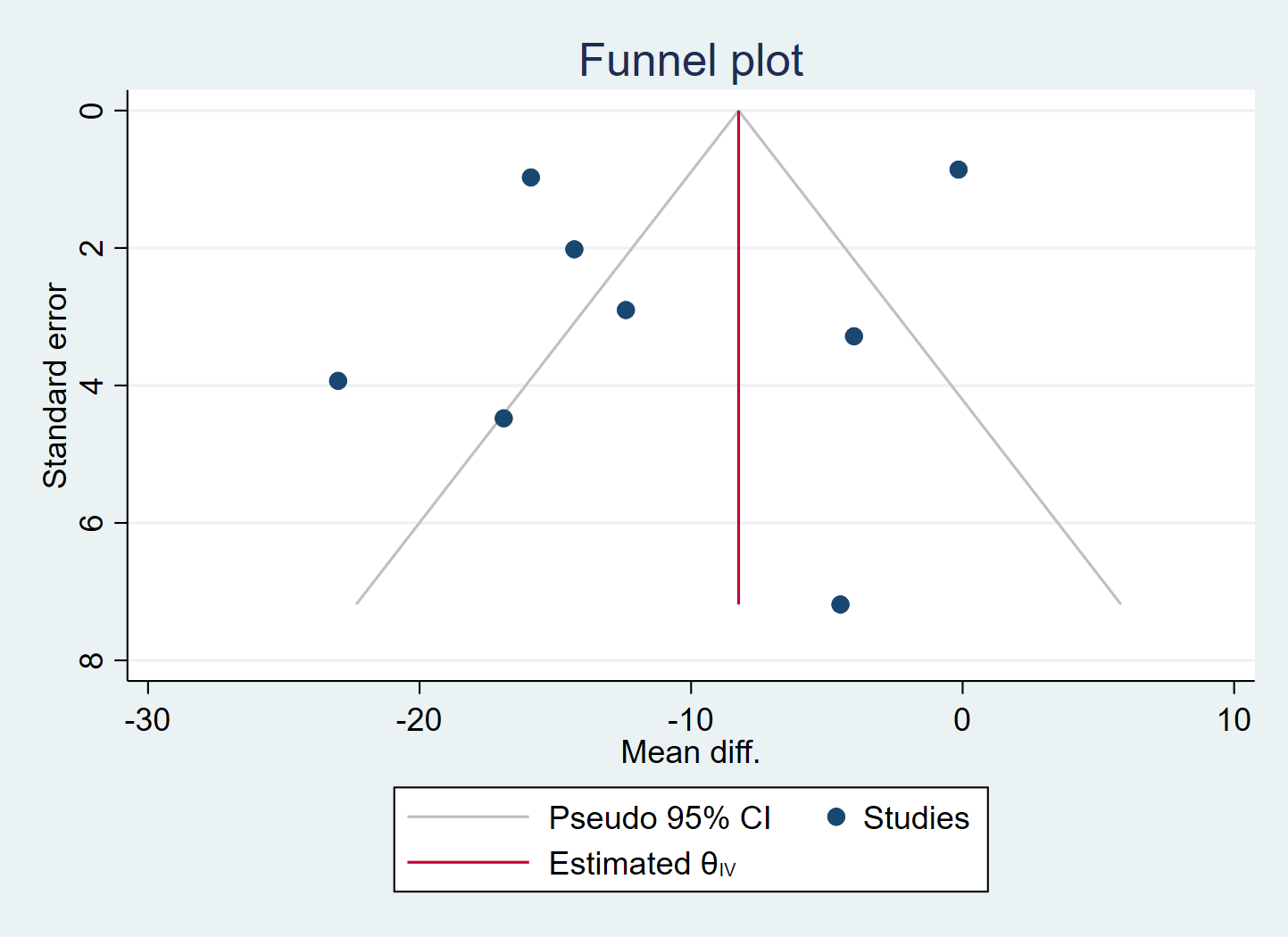
(D)
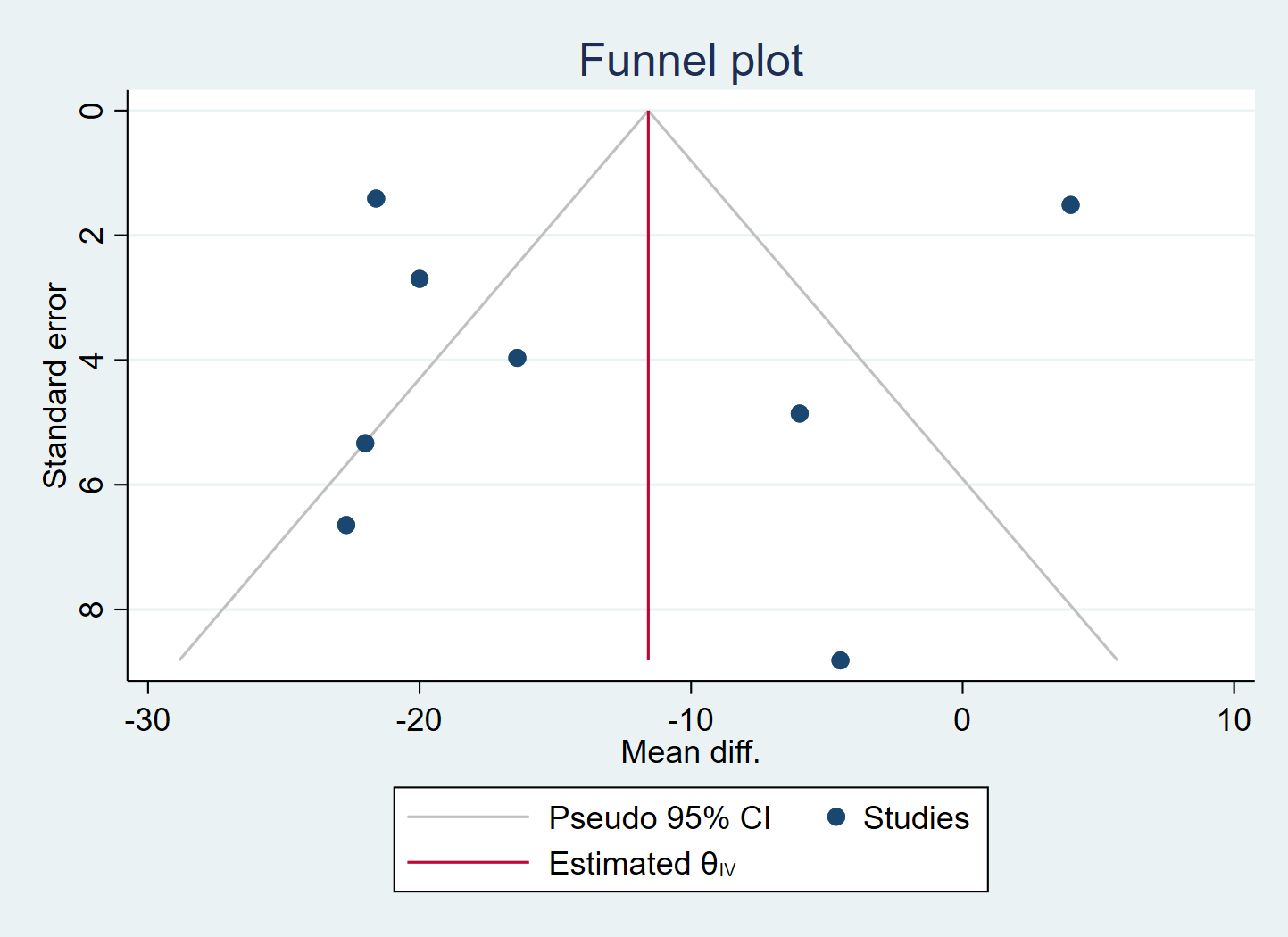
(E)
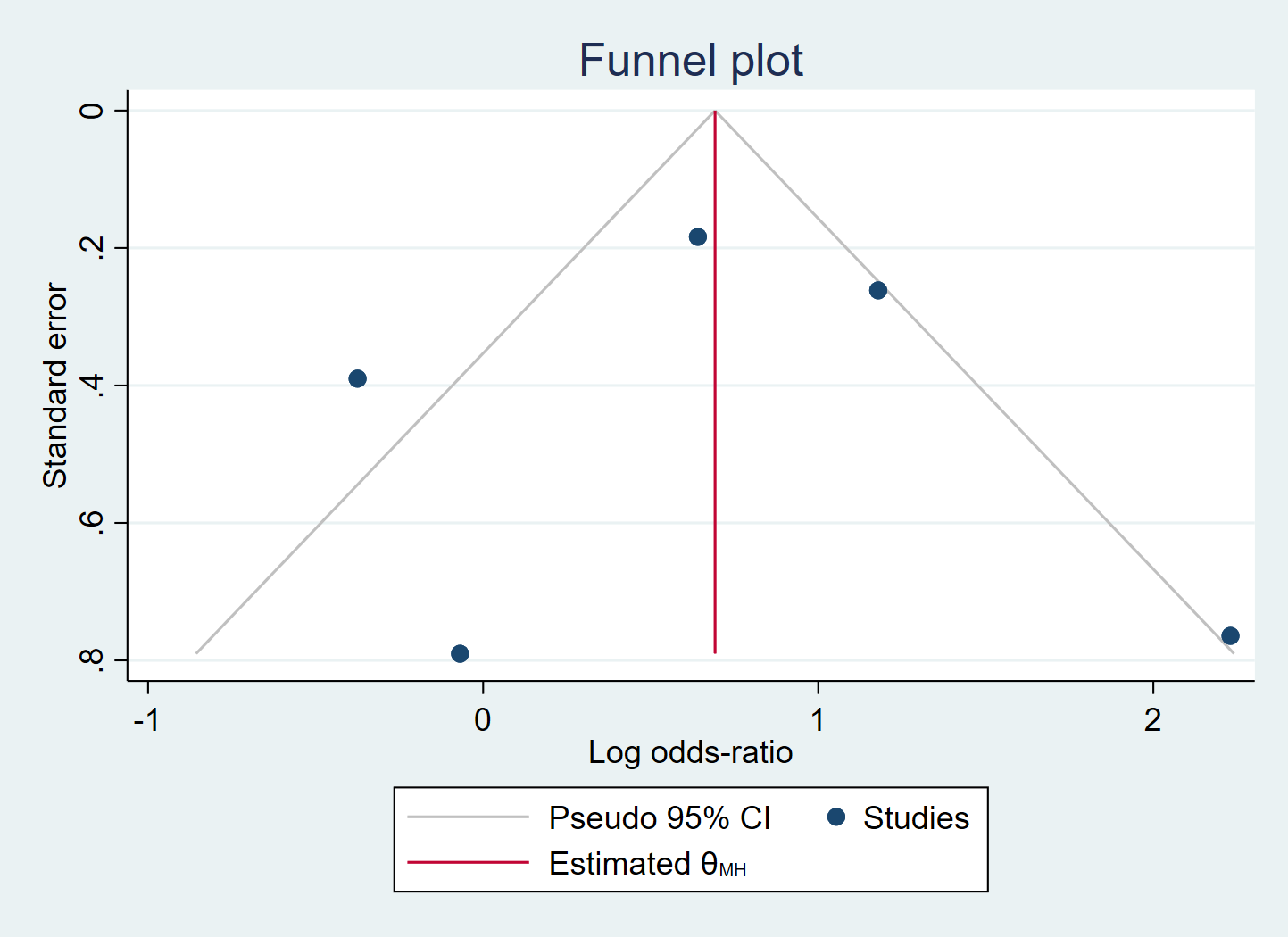
(F)
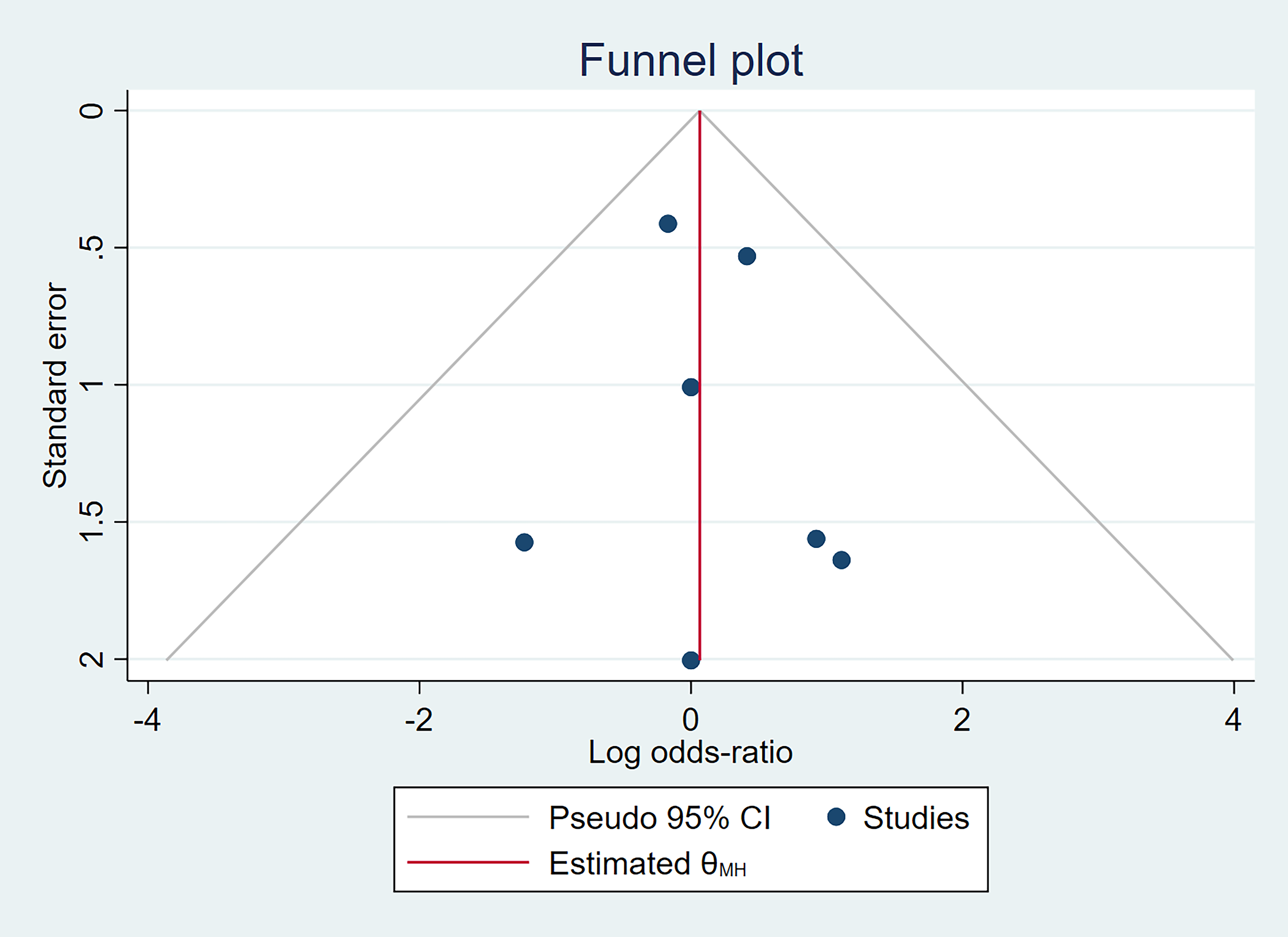
(G)
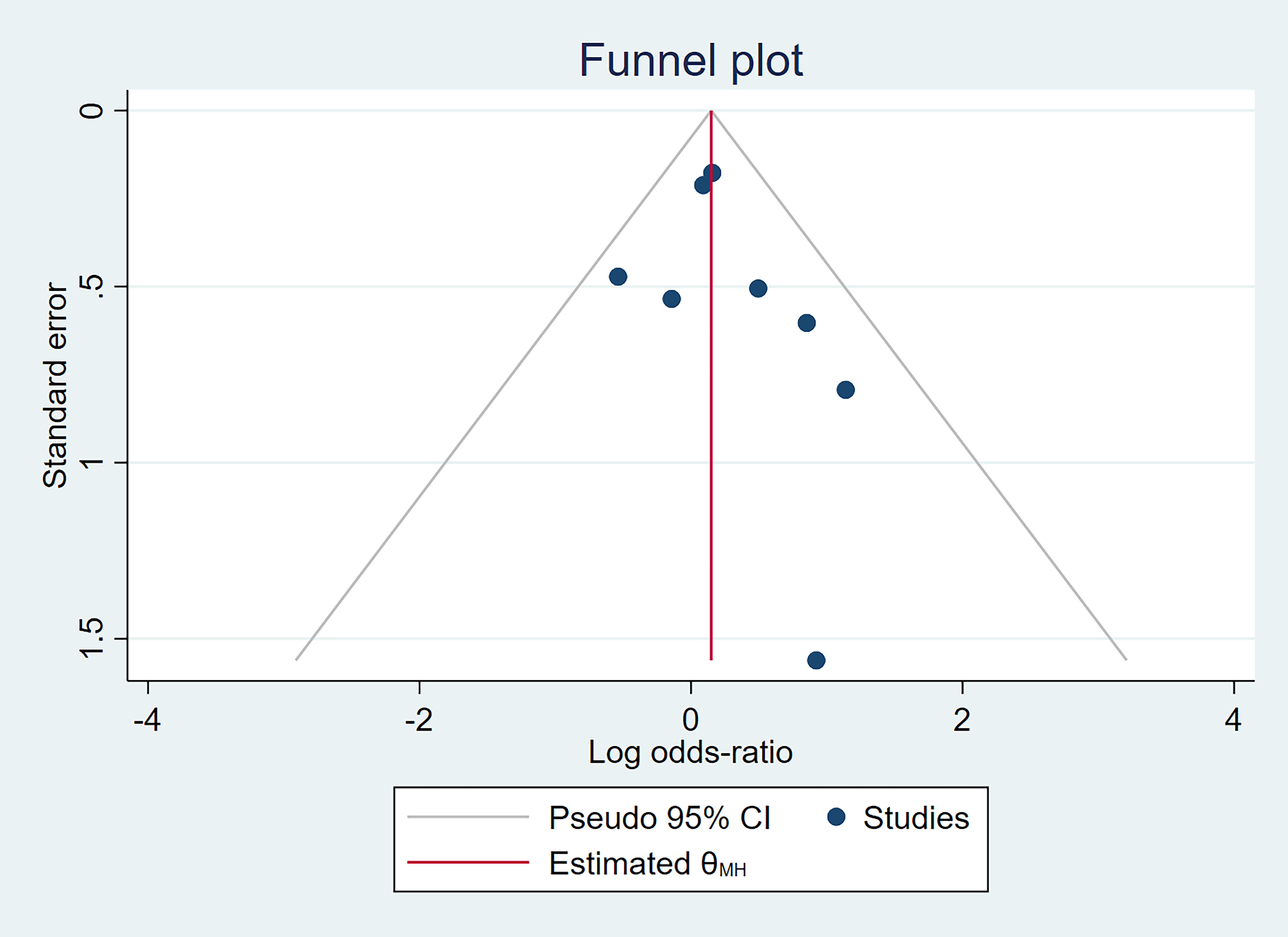
(H)
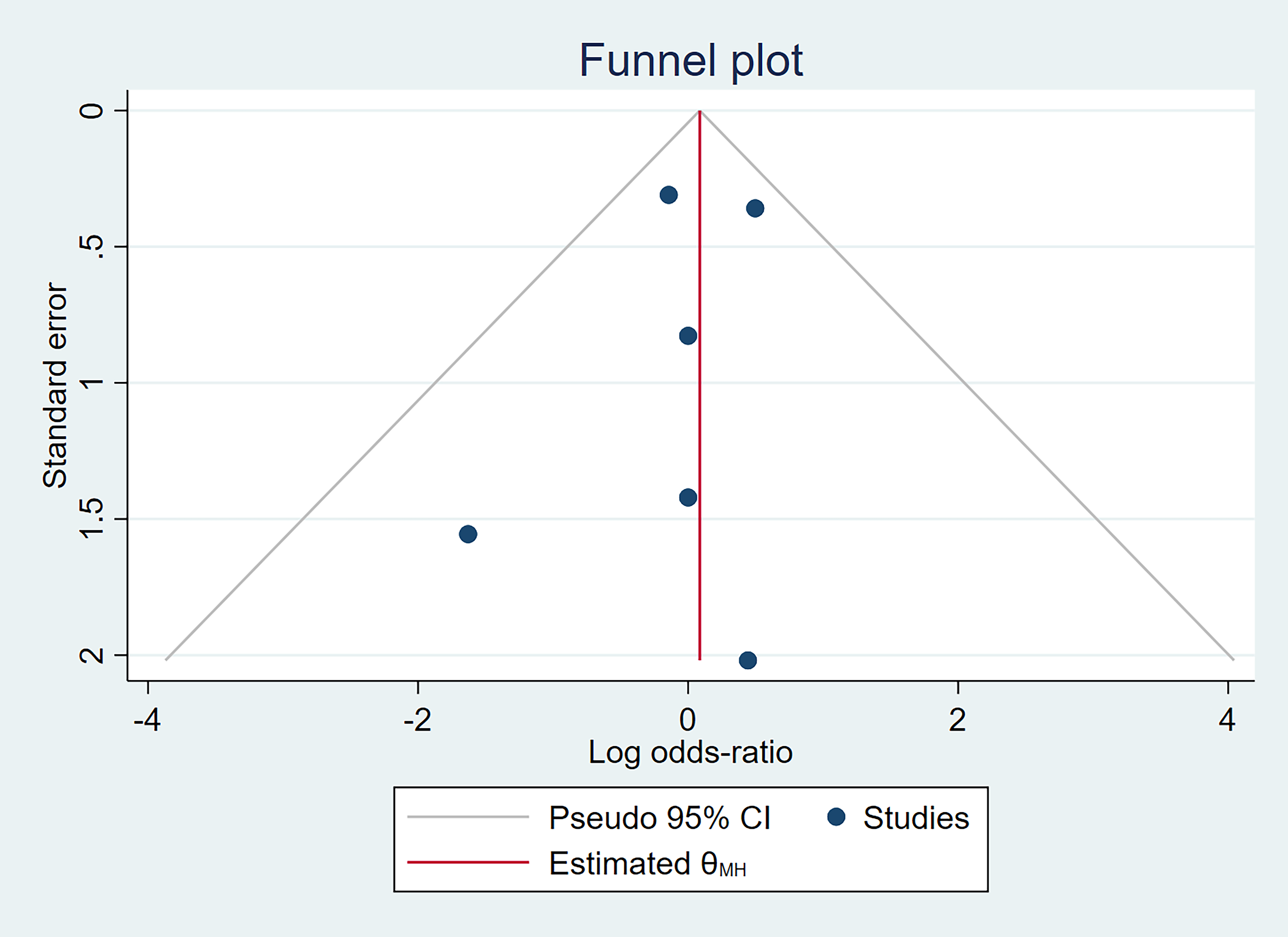
(I)
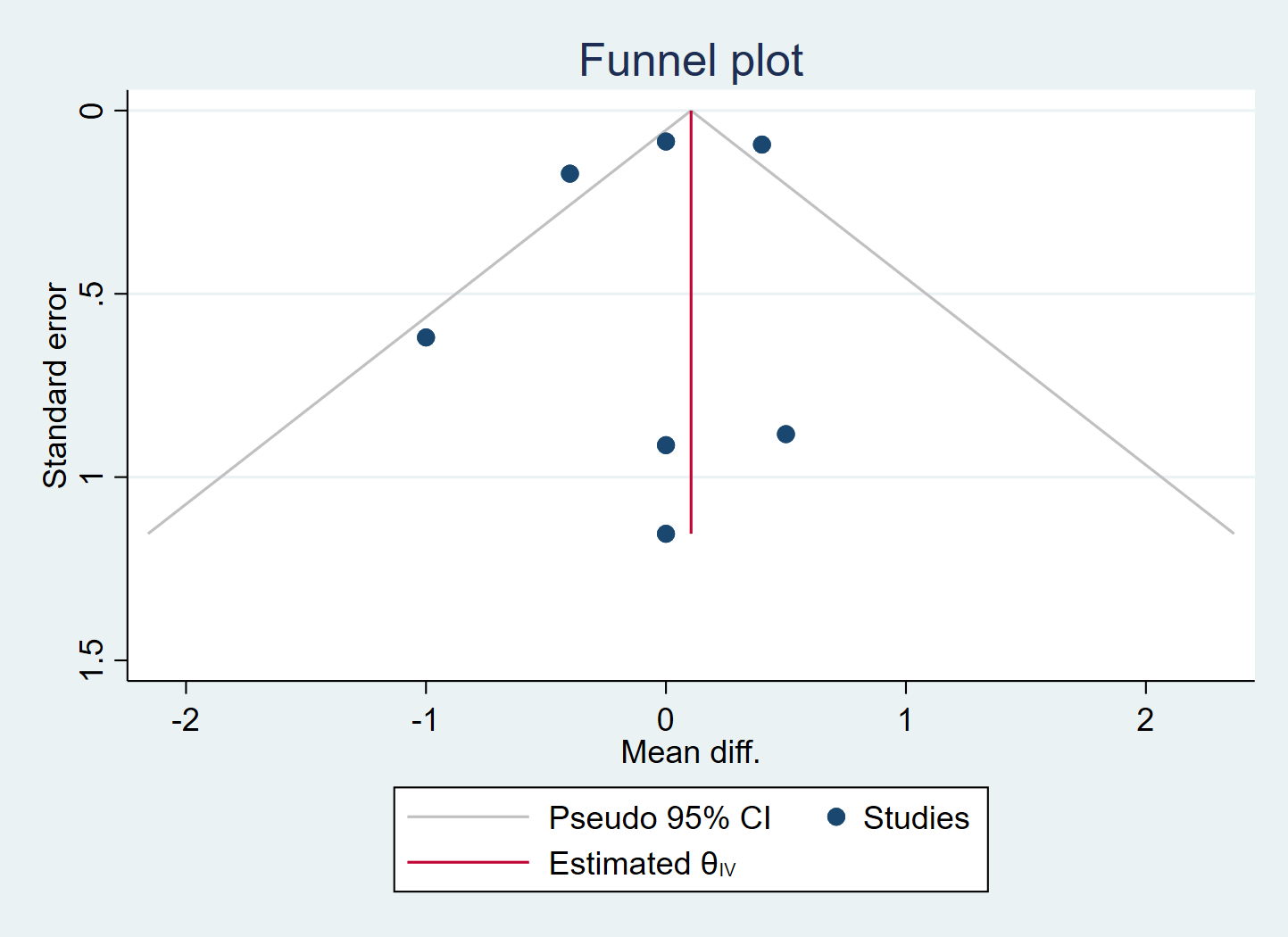
(J)

**Figure S5:** Funnel plots from STATA16.0. (A) early mortality; (B) mean pressure gradients; (C) peak pressure gradients; (D) aortic cross-clamp time; (E) cardiopulmonary time; (F) any paravalvular leak; (G) moderate-severe paravalvular leak; (H) pacemaker implantation; (I) stroke; (J) ICU-stay

Supplementary tables caption:

**
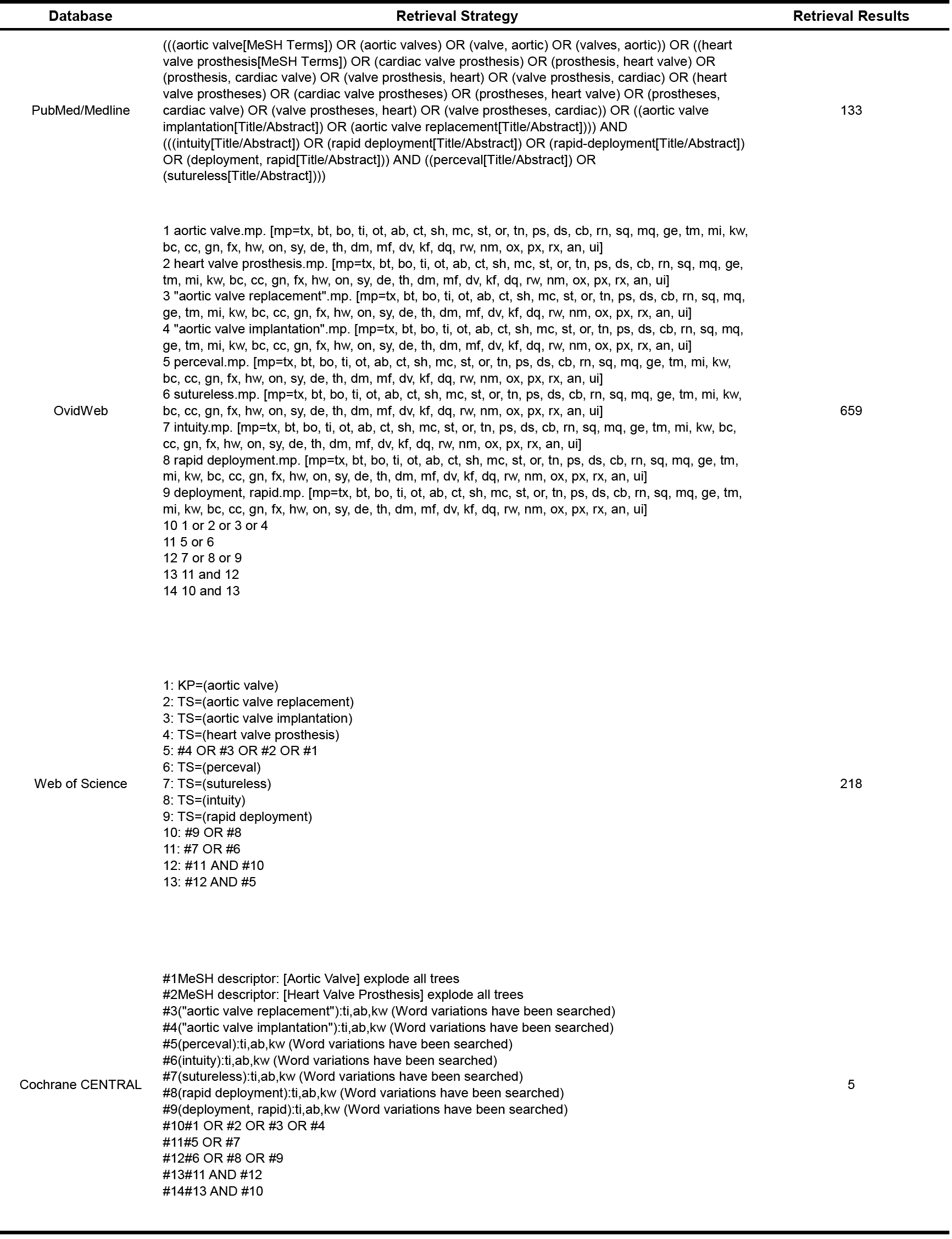
Table S1**: Electronic Database Retrieval Strategy. MEDLINE = Medical Literature Analysis and Retrieval System Online; CENTRAL = Central Register of Controlled Trials.

**
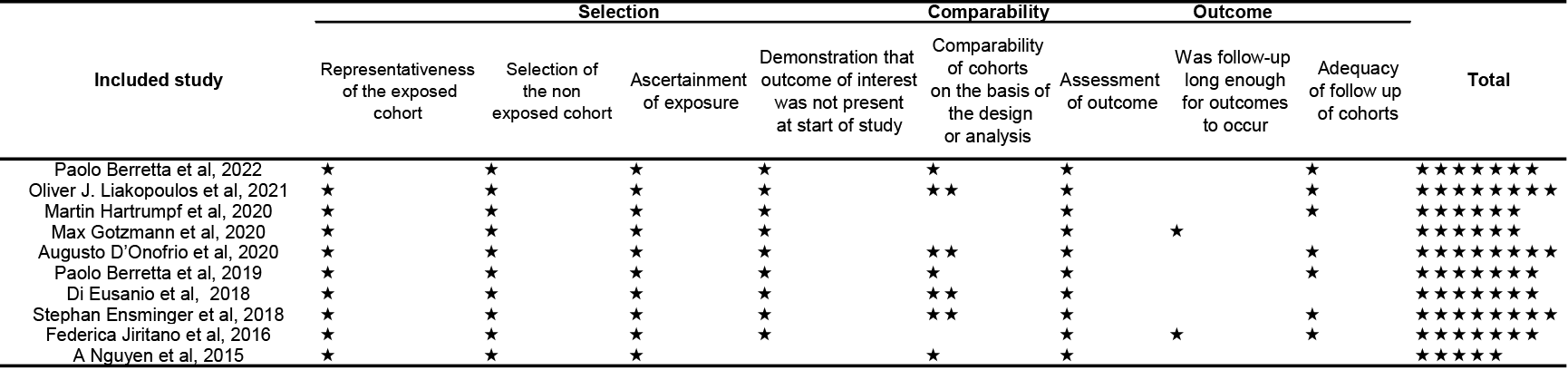
Table S2:** Quality Assessment by NEWCASTLE-OTTAWA SCALE (NOS) for observational study.
