# Supplementary figures and images for: Sutureless vs. rapid-deployment valve: a systemic review and meta-analysis for a direct comparison of intraoperative performance and clinical outcomes
Source: Front Cardiovasc Med. 2023 May 15;10:1123487. doi: 10.3389/fcvm.2023.1123487 (PMC10225698; doi:10.3389/fcvm.2023.1123487)

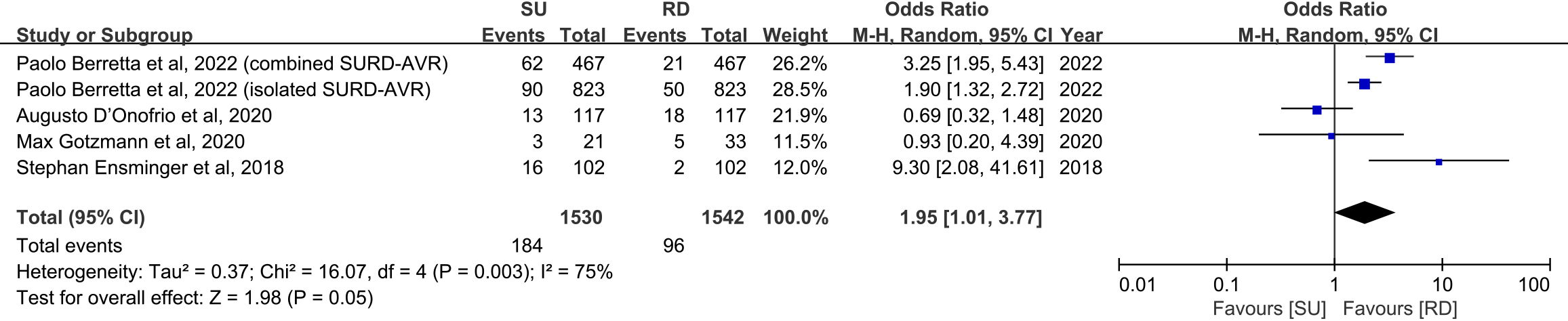

Supplement: Supplementary file 2 [file Image1.tif]

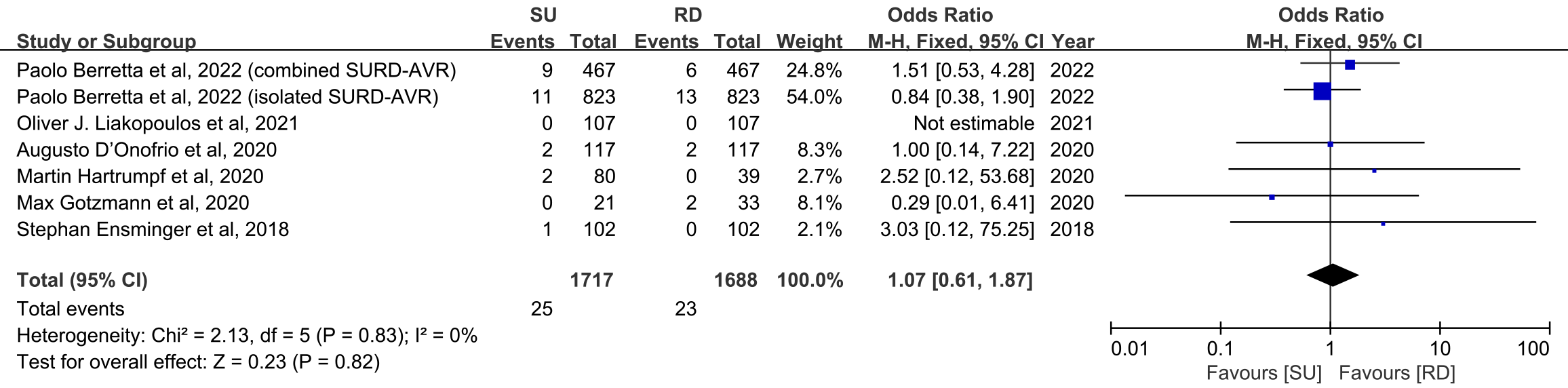

Supplement: Supplementary file 3 [file Image2.tif]

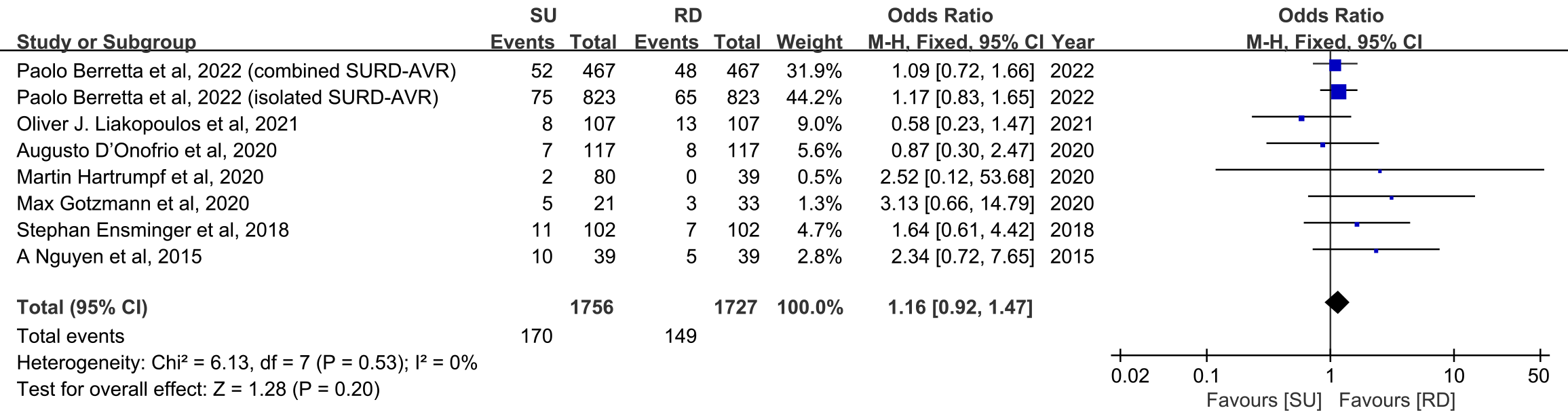

Supplement: Supplementary file 4 [file Image3.tif]

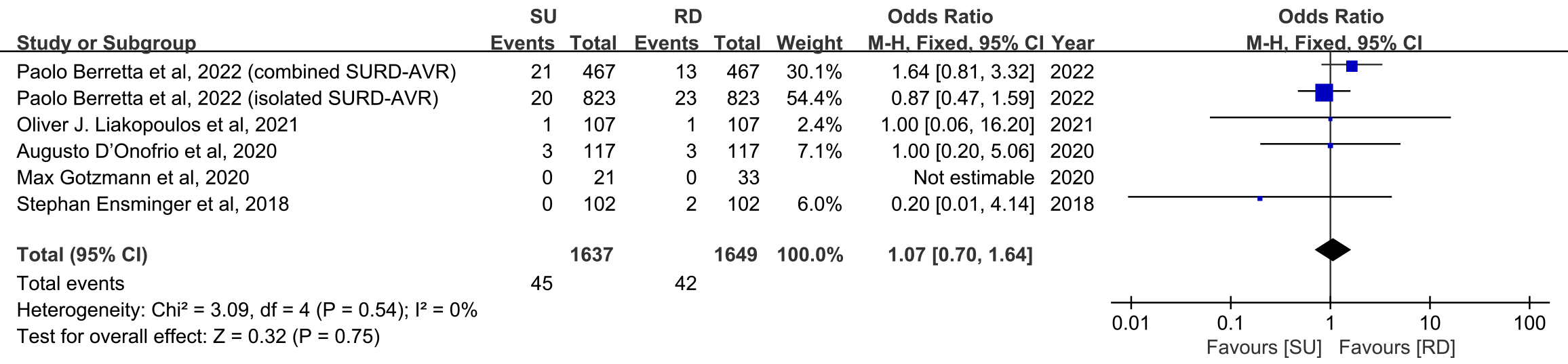

Supplement: Supplementary file 5 [file Image4.tif]

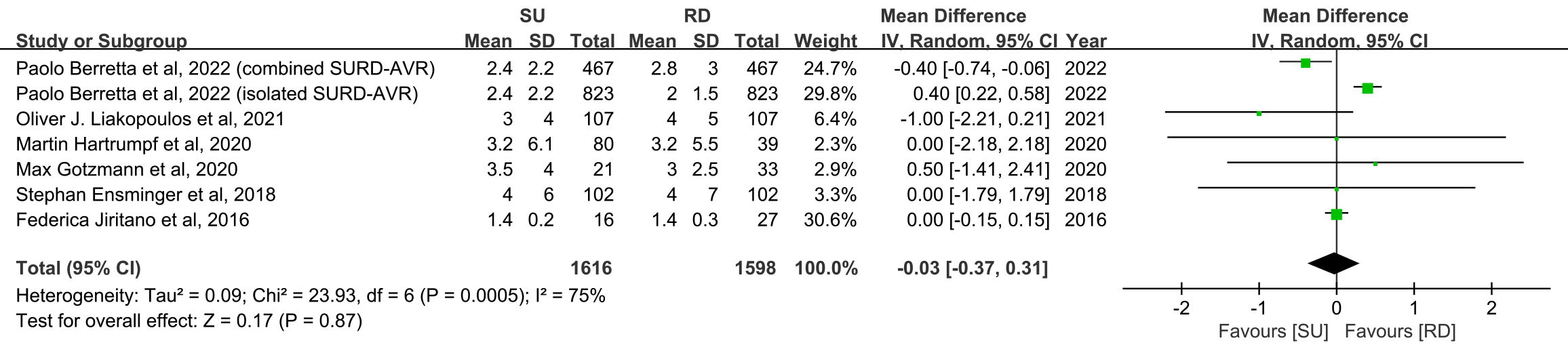

Supplement: Supplementary file 6 [file Image5.tif]

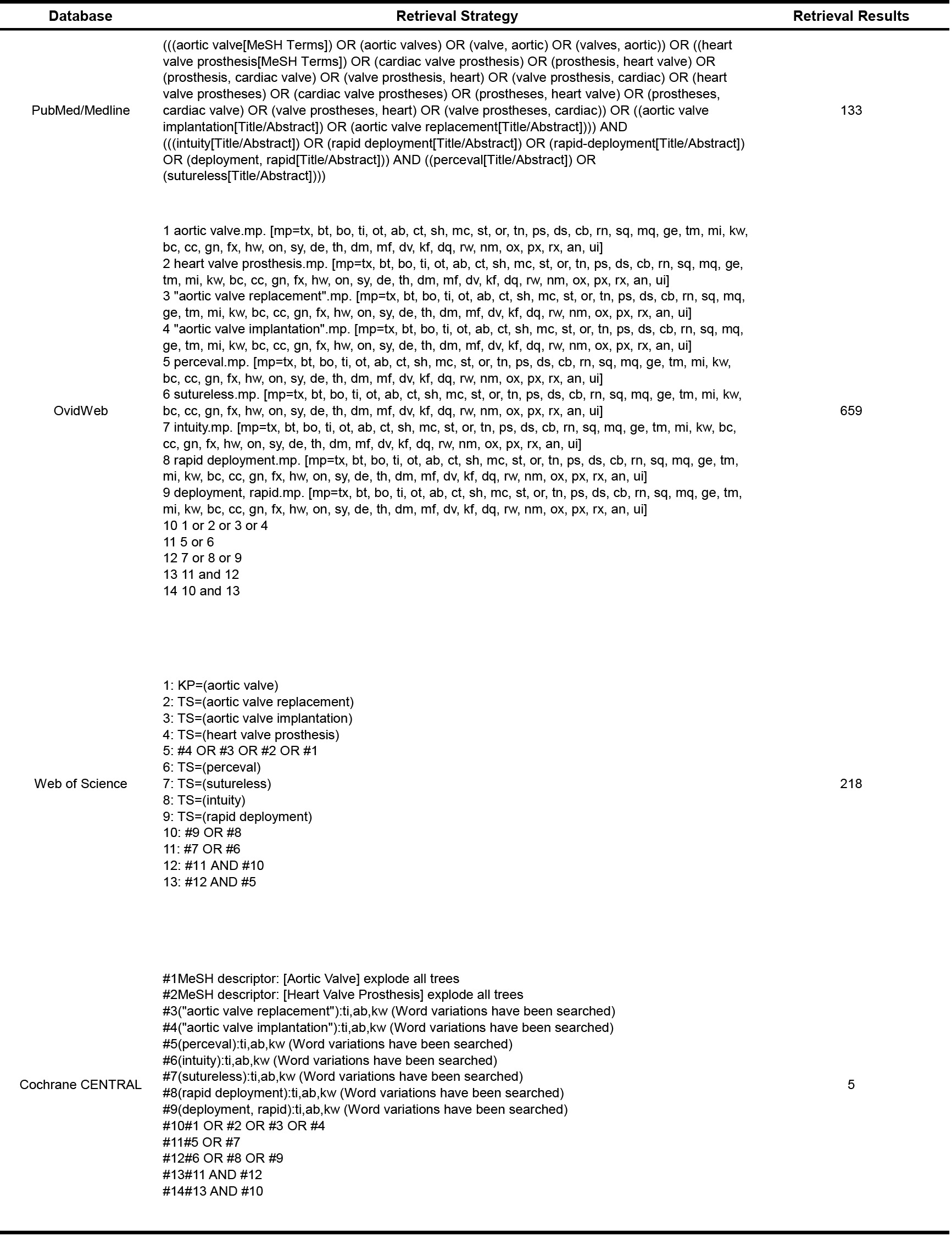

Supplement: Supplementary file 7 [file Image6.tif]

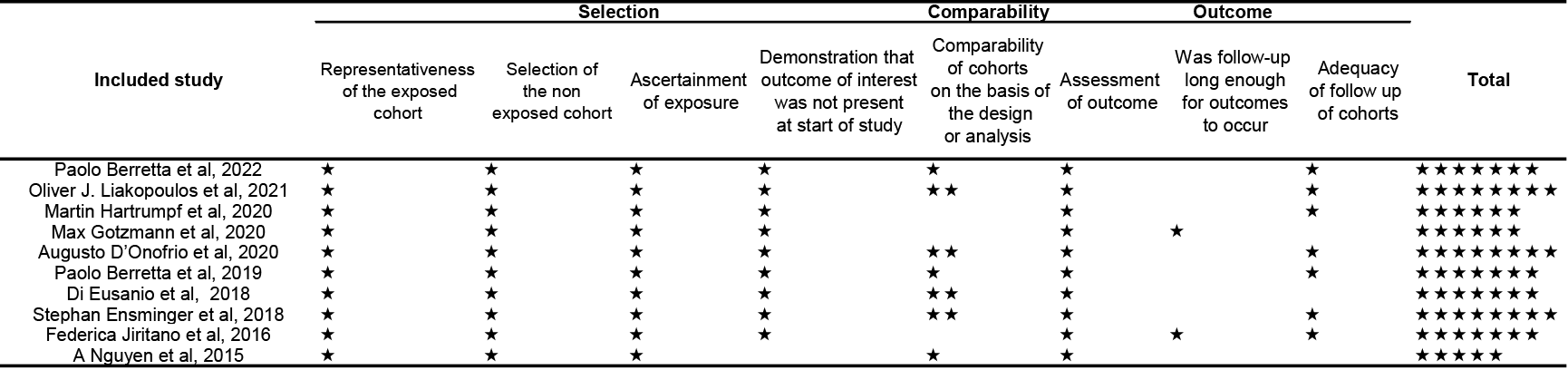

Supplement: Supplementary file 8 [file Image7.tif]
